# Engineered extracellular vesicles enable high-efficient delivery of intracellular therapeutic proteins

**DOI:** 10.1093/procel/pwae015

**Published:** 2024-03-22

**Authors:** Ding Ma, An Xie, Jiahui Lv, Xiaolin Min, Xinye Zhang, Qian Zhou, Daxing Gao, Enyu Wang, Lei Gao, Linzhao Cheng, Senquan Liu

**Affiliations:** Department of Hematology, The First Affiliated Hospital of USTC, Division of Life Sciences and Medicine, University of Science and Technology of China, Hefei 230001, China; Blood and Cell Therapy Institute, Anhui Provincial Key Laboratory of Blood Research and Applications, University of Science and Technology of China, Hefei 230036, China; School of Basic Medical Sciences, Division of Life Sciences and Medicine, University of Science and Technology of China, Hefei 230027, China; Blood and Cell Therapy Institute, Anhui Provincial Key Laboratory of Blood Research and Applications, University of Science and Technology of China, Hefei 230036, China; School of Biomedical Engineering, Division of Life Sciences and Medicine, University of Science and Technology of China, Hefei 230026, China; School of Basic Medical Sciences, Division of Life Sciences and Medicine, University of Science and Technology of China, Hefei 230027, China; School of Basic Medical Sciences, Division of Life Sciences and Medicine, University of Science and Technology of China, Hefei 230027, China; School of Basic Medical Sciences, Division of Life Sciences and Medicine, University of Science and Technology of China, Hefei 230027, China; School of Basic Medical Sciences, Division of Life Sciences and Medicine, University of Science and Technology of China, Hefei 230027, China; School of Basic Medical Sciences, Division of Life Sciences and Medicine, University of Science and Technology of China, Hefei 230027, China; Blood and Cell Therapy Institute, Anhui Provincial Key Laboratory of Blood Research and Applications, University of Science and Technology of China, Hefei 230036, China; Blood and Cell Therapy Institute, Anhui Provincial Key Laboratory of Blood Research and Applications, University of Science and Technology of China, Hefei 230036, China; Department of Hematology, The First Affiliated Hospital of USTC, Division of Life Sciences and Medicine, University of Science and Technology of China, Hefei 230001, China; Blood and Cell Therapy Institute, Anhui Provincial Key Laboratory of Blood Research and Applications, University of Science and Technology of China, Hefei 230036, China; School of Basic Medical Sciences, Division of Life Sciences and Medicine, University of Science and Technology of China, Hefei 230027, China; School of Biomedical Engineering, Division of Life Sciences and Medicine, University of Science and Technology of China, Hefei 230026, China; Department of Hematology, The First Affiliated Hospital of USTC, Division of Life Sciences and Medicine, University of Science and Technology of China, Hefei 230001, China; Blood and Cell Therapy Institute, Anhui Provincial Key Laboratory of Blood Research and Applications, University of Science and Technology of China, Hefei 230036, China; School of Basic Medical Sciences, Division of Life Sciences and Medicine, University of Science and Technology of China, Hefei 230027, China

**Keywords:** extracellular vesicles, intracellular protein delivery, cGAS, cancer immunotherapy

## Abstract

Developing an intracellular delivery system is of key importance in the expansion of protein-based therapeutics acting on cytosolic or nuclear targets. Recently, extracellular vesicles (EVs) have been exploited as next-generation delivery modalities due to their natural role in intercellular communication and biocompatibility. However, fusion of protein of interest to a scaffold represents a widely used strategy for cargo enrichment in EVs, which could compromise the stability and functionality of cargo. Herein, we report intracellular delivery via EV-based approach (IDEA) that efficiently packages and delivers native proteins both *in vitro* and *in vivo* without the use of a scaffold. As a proof-of-concept, we applied the IDEA to deliver cyclic GMP-AMP synthase (cGAS), an innate immune sensor. The results showed that cGAS-carrying EVs activated interferon signaling and elicited enhanced antitumor immunity in multiple syngeneic tumor models. Combining cGAS EVs with immune checkpoint inhibition further synergistically boosted antitumor efficacy *in vivo*. Mechanistically, scRNA-seq demonstrated that cGAS EVs mediated significant remodeling of intratumoral microenvironment, revealing a pivotal role of infiltrating neutrophils in the antitumor immune milieu. Collectively, IDEA, as a universal and facile strategy, can be applied to expand and advance the development of protein-based therapeutics.

## Introduction

The broad application of protein and gene therapeutics requires safe and efficient methods for delivery to diverse tissues and organs (Banskota et al., 2022; Kreitz et al., 2023; Morshedi Rad et al., 2021; Segel et al., 2021; Stewart et al., 2016). To overcome the hurdle of delivery, substantial efforts have been made to take advantage of both viral and nonviral vectors (Ling et al., 2021; Stewart et al., 2016; Tian et al., 2022). The major shortcomings of viral vectors, such as adenoviruses/adeno-associated viruses and retroviruses/lentiviruses, include immunogenicity and insertional mutagenesis, as well as challenges in large-scale production (Bulcha et al., 2021; Earley et al., 2023; Kasala et al., 2021; Milone and O’Doherty, 2018; Shirley et al., 2020). On the other hand, non-viral vectors including synthetic polymers and lipid nanoparticles (LNPs), have been extensively investigated. However, limited circulation, biodistribution, and low efficiency limit the use of these synthetic delivery systems in many therapeutic applications (Hou et al., 2021; Islam et al., 2018; Lokugamage et al., 2021; Machtakova et al., 2022; Paunovska et al., 2022; Vargason et al., 2021; Yang et al., 2023; Zong et al., 2023).

It is widely accepted that the direct delivery of therapeutic proteins could circumvent some issues associated with the gene transfer of DNAs and RNAs (Liu et al., 2018; Qin et al., 2019; Sanchez-Navarro, 2021). The safety as well as efficacy of therapeutic proteins including intracellular enzymes, transcription factors, or gene editors such as CRISPR/Cas, often need their actions to be transiently and temporally controlled. Additionally, proteins can initiate intracellular activities without transcription/translation delays (Stewart et al., 2016). Intracellular delivery of bioactive proteins to replace missing, dysfunctional, or poorly expressed proteins or antagonize key intracellular pathways is the fastest-growing and promising approach in modern drug development. Protein-based biologics have provided new therapeutic avenues for the treatment of a range of diseases, including cancer, inflammation, and degeneration. In addition to therapeutics, direct cytosolic or nuclear delivery of functional proteins provides a potential tool for important biological applications, including imaging, signaling studies, and other forms of cell engineering. Nonetheless, a major limitation to using proteins as intracellular payloads is the current lack of technologies that can introduce therapeutic proteins into the cytosol or nucleus of cells after crossing plasma membrane, with both high efficiency and low toxicity (Gouveia et al., 2023; Ren et al., 2023).

Extracellular vesicles (EVs), which are membrane-encapsulated and nano-sized particles, have emerged as promising carriers for delivering bioactive cargos owing to their intrinsic biocompatibility, low immunogenicity, and great capability to protect luminal contents from degradation and cross physiological barriers (Escude Martinez de Castilla et al., 2021; Gao et al., 2023; Sheller-Miller et al., 2021; Yom-Tov et al., 2022; You et al., 2023). To improve the efficacy of EVs for protein-based therapies, it would be even better to produce EVs containing a specific type of therapeutic proteins that are synthesized in producing cells and encapsulated in EVs for better protection and delivery into recipient cells. The current standard strategy is to fuse the protein of interest to a scaffold protein/peptide for improved cargo sorting and enrichment in EVs. However, protein fusion often compromises the stability and functionality of proteins (Choi et al., 2020; Dooley et al., 2021; Ilahibaks et al., 2023; Silva et al., 2021; Zhang et al., 2020; Zheng et al., 2023). Moreover, existing EV-mediated strategies thus far support only modest efficiencies with limited validation of therapeutic efficacy *in vivo*, especially for intercellular targets, as being pointed out by two recent expert reviews (Cecchin et al., 2023; Escude Martinez de Castilla et al., 2021). To address this problem, we devised a simple and reliable functional assay to optimize EV cargo loading and to generate engineered EVs for efficient delivery of nontethered bioactive proteins to cytosolic or nuclear compartments. Herein, we describe the development and application of intracellular delivery of bioactive proteins via EV-based approach (IDEA) for packaging and delivering bioactive proteins without the use of a scaffold protein, thereby offering key advantages compared with both viral and synthetic delivery strategies.

## Results

### Design of a novel platform for high-efficient intracellular delivery of bioactive proteins

To systematically investigate the EV-mediated intracellular delivery of bioactive proteins, we devised a reporter assay based on Cre-*loxP* recombination (Fig. S1A). In recipient cells, two *loxP* sites flank a fluorescent reporter gene (*DsRed*) with a stop codon. Once the functional Cre protein is present in nucleus of EV-treated recipient cells, it will activate DNA recombination between two *loxP* sites and excise the intervening DNA sequence including the *DsRed* and a translational stop codon, leading to normal GFP translation and production (Fig. S1A). The HEK293F cell line was selected as EV-producing cells based on its well-established documentation of safety, the ease of gene manipulation with high efficiency, the possibility of culture in a chemically defined medium devoid of contaminating EVs from animal serum, and the capacity to give more than 10-fold EV yield compared to other primary or immortalized cells (Dooley et al., 2021). To confirm whether EVs carry Cre proteins, EVs were isolated from the culture medium of Cre-expressing cells 72 h after transient transfection of expression vectors. EVs were purified through ultracentrifugation followed by size-exclusion chromatography (SEC) (Fig. S1B). Encouraged by recent developments in engineered virus-like particles (eVLPs) for intracellular delivery (Banskota et al., 2022; Hamilton et al., 2021), we also explored if adding some vial genes, such as retroviral gag-pol and vesicular stomatitis virus G envelope glycoprotein (VSV-G) could also enhance the payload in our engineered EV system in comparison with eVLP. The same Cre-*loxP* recombination recipient cells are also used to validate the eVLP system for Cre recombinase delivery (Fig. S1A). To this end, we expressed Cre as a fusion to the gag which is essentially a membrane-anchored scaffold protein as previously reported. We produced eVLPs using the strategy similar to EVs (Fig. S1B). We successfully validated the packaging and delivery of Cre recombinase-mediated by eVLPs produced from HEK293F cells transfected with three constructs expressing gag-Cre as well as gag-pol and VSV-G (Fig. S1C). Unexpectedly, we found that the gag-pol encoding a retroviral structure protein was not necessary for successful packaging and delivery, although it modestly improved the efficiency of gene modification in the GFP reporter cells (Fig. S1C). Hence, gag-pol was first withdrawn and we called the vesicles without gap-pol as engineered EVs (eEVs), versus the eVLPs that were produced with gag-pol. Produced by co-transfection with gag-Cre and VSV-G, eEVs exhibited Cre-mediated reporter efficiencies similar to eVLPs, in a dose-dependent manner (Fig. S1D). However, we noted that eVLPs or eEVs expressing gag-Cre mediated DNA recombination is sub-optimal (~15% GFP^+^ cells), which is less efficient than direct DNA transfection of the gag-Cre vector to the same reporter cells (Fig. S1E).

**Figure 1.**
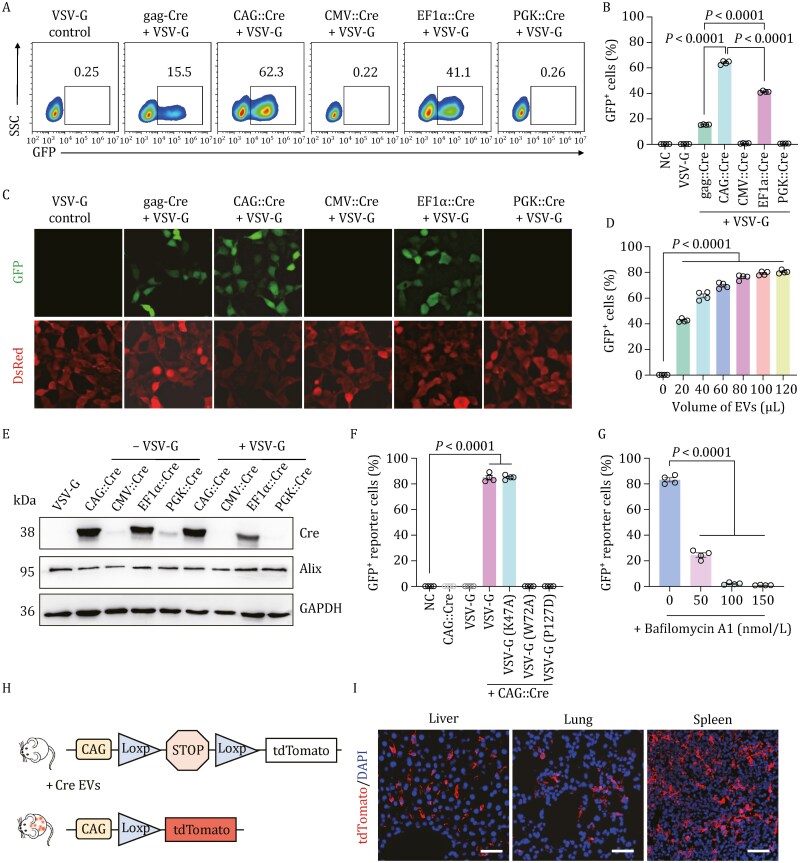
An engineered EV-mediated delivery platform (IDEA) enables high-efficient intracellular transfer of functional proteins. (A) Representative flow cytometric data comparing the efficiency of packaging and delivery of Cre recombinase through VSV-G packaged EVs. (B) Quantification of GFP^+^ recipient cells after engineered EV incubation for 48 h. (C) Fluorescence images of reporter cells treated with engineered EVs carrying Cre recombinase. Scale bar, 20 μm. (D) Cre delivery efficiencies of various amounts of engineered EVs produced with CAG::Cre vector. (E) Western blot analysis of the cargo protein Cre and the classical EV marker Alix in purified EVs. (F) Reporter cells were treated with EVs displaying wildtype VSV-G or mutant VSV-G (K47A, W72A or P127D). (G) Engineered EVs with VSV-G were applied to recipient cells in the presence of 10, 50 or 100 nmol/L bafilomycin A1. (H) Schematic diagram of measuring *in vivo* Cre-loxP recombination using Ai14 mice. Excision by Cre results in tdTomato production in various organs. (I) Representative immunofluorescence images of expressed tdTomato over nuclear (DAPI) staining from tissue sections of selected organs that were collected from Ai14 mice intraperitoneally administered with Cre EVs. Scale bar, 50 μm. For all quantifications, data are displayed as mean ± SEM from four biological replicates. Statistical analyses were performed using a two-sided unpaired *t*-test.

We reasoned that the transgene expression level and protein abundance in producer cells could also be a bottleneck of Cre cargo packaging in EVs. To test this hypothesis, we pre-screened a panel of transcriptional enhancers/promoter units driving Cre expression (Fig. S2A). Encouragingly, CAG::Cre EVs exhibited the highest delivery efficiencies compared to eVLPs when externally administered to target cells (Fig. 1A and 1B). More importantly, the Cre activity delivered by EVs was no longer dependent on the gag fusion once VSV-G was co-expressed; EVs produced with a CAG::Cre or EF1a::Cre vector exhibited a high-level of GFP induction (40%–65%) as shown in Fig. 1A and 1B. The result was further confirmed by direct imaging of GFP signal (Fig. 1C). Strikingly, CAG::Cre EVs robustly achieved an efficiency of > 80% GFP^+^ cells, in a dose-dependent manner (Fig. 1D). Together, the high-level activity of CAG-EVs no longer requires the presence of gag-pol (Fig. S2B). The high-level of Cre proteins was found in both EVs (Fig. 1E) as well as their producer cells (Fig. S2C), when CAG (and EF1a to a less extent) was used to drive Cre Expression. However, only the co-expression of VSV-G with CAG::Cre in EV production achieved high-level of GFP^+^ cells in the Cre-*loxP* reporter cells (~80%), as compared to almost zero when CAG::Cre was expressed alone (Figs. 1F and  S2B). We called this optimized strategy as IDEA, in which the viral gag-pol or fusion of the cargo to gag is no longer necessary, but co-expression of VSV-G is required for efficient delivery.

We next investigated the critical roles of VSV-G in EV-mediated Cre delivery. VSV-G as an envelope protein is widely used for packaging recombinant retrovirus/lentivirus to achieve gene transduction successfully. It is believed that VSV-G is involved in receptor recognition at the host cell surface and then, after endocytosis of the virion, triggers membrane fusion via a low pH-induced structural rearrangement (Beilstein et al., 2020; Finkelshtein et al., 2013; Nikolic et al., 2018). We reasoned the enhancing effect of VSV-G on EV-mediated Cre delivery could be due to (i) enhanced membrane docking and entry of EVs into recipient cells; (ii) improved endosomal/lysosomal escape after uptake after endocytosis. We initiated investigation to determine the major roles of VSV-G in our IDEA delivery system. We observed an approximately 2-fold increase in production of EVs by VSV-G co-transfection in producing cells, without changing the size distribution of released EVs (data not shown). We next tested co-transfection of VSV-G variants with defects in different aspects of VSV-G functions. The VSV-G mutant K47A, which lacks the binding activity to the LDL receptor family on cell-surface (Nikolic et al., 2018), did not show a defect in EV-mediated Cre activity (Fig. 1F). This suggests that VSV-G is likely acting on other downstream steps beyond the docking to target cell surface. Indeed, the EVs expressing VSV-G mutant W72A or P127D that lost the membrane fusion ability (Votteler et al., 2016) resulted in little Cre-mediated activity to generate GFP^+^ cells, as compared to the EVs harboring wild-type VSV-G or K47A mutant (Fig. 1F). These data strongly support that VSV-G enhanced cargo delivery of Cre EVs to nucleus is likely through the membrane fusion and somehow disrupting the trapping and degradation of membraned organelles such as endosomes and lysosomes. To further test this hypothesis, we applied a small cell-permeable molecule bafilomycin A1 which is known to prevent the acidification of endosomes/lysosomes and then disrupts their functions (Yonezawa et al., 2005). In the presence of this inhibitor, enhancement of Cre recombination by VSV-G was almost completely abolished in reporter cells (Fig. 1G), without dramatically affecting cell viability and EV internalization (data not shown). Together, the results suggest that VSV-G-mediated membrane fusion (likely through escape of endosomal/lysosomal degradation) is indispensable for the successful delivery of EV cargos to intracellular compartments.

Having confirmed the capability of the IDEA platform to successfully deliver intracellular proteins *in vitro*, the *in vivo* applicability of engineered EVs was next assessed (Fig. 1H). To ascertain whether engineered EVs could deliver functional Cre recombinase *in vivo*, we intraperitoneally administered highly purified EVs carrying Cre recombinase to Ai14 reporter mice at 1 × 10^12^ EVs for two treatments (Fig. 1H). Control Ai14 mice were treated with PBS or control EVs. These mice possess a LoxP-STOP-LoxP insertion in the Gt (ROSA)26Sor locus, upstream of the reporter gene that encodes the tandem dimer Tomato (tdTomato). In the presence of EV-delivered Cre recombinase, the STOP codon shall be excised, resulting in tdTomato production in recipient cells. One week after EV administration, the organs (liver, lung, spleen, heart, kidney, brain, and intestine) were harvested and stained for tdTomato. The results indicated that Cre-EVs entered into a substantial number of cells in the liver, lung, and spleen, and activated the nuclear DNA recombination *in vivo*, following systemic administration (Figs. 1H, 1I and S2D). Furthermore, EV injection did not lead to toxicity in the organs, as evidenced by normal morphology (data not shown). Combined, our findings demonstrate that engineered EVs can efficiently package cargo proteins such as Cre, enter recipient cells to achieve intracellular delivery *in vitro* and *in vivo*.

To confirm that the utility of IDEA platform is not restricted to gene modification enzymes, we assessed this optimized platform for the delivery of β-catenin, a cytosolic protein that would translocate to nucleus and work as a transcriptional co-factor after Wnt activation. It is well known that compromised regeneration resulting from the deactivation of Wnt/β-catenin signaling contributes to the progression of numerous diseases with limited therapeutic options (Nusse and Clevers, 2017; Russell and Monga, 2018; Schunk et al., 2021). Given that the hydrophobic nature of Wnt proteins limits their purification and use (Routledge and Scholpp, 2019; Wolf and Boutros, 2023), intracellular transfer of the Wnt signal transducer β-catenin holds great promise. We applied IDEA strategy to produce EVs by co-expressing β-catenin and VSV-G together, and purified EVs were generated as described above. The presence of β-catenin proteins in EVs as well as in the producer cells was verified by immunoblotting (Fig. S2E). To further confirm that the β-catenin protein delivered by IDEA is biologically functional, a TOPFlash reporter assay was applied (Fig. S2F). As expected, β-catenin-loaded EVs obtained from IDEA led to a significant induction of luciferase activity in the reporter cells (Fig. S2G). Using two distinct functional assay systems, we demonstrated that intracellular proteins can be efficiently expressed and packaged in EVs, and delivered successfully to nucleus using our IDEA platform.

### Targeted delivery of cGAS by IDEA activates innate immune signaling *in vitro*

To broaden the therapeutic applications of IDEA, we commenced assessing its capability for cancer immunotherapy by delivering cGAS, a key intracellular immune regulator. Over the past years, manipulation of the cGAS-STING pathway has gained much interest in immune oncology (Samson and Ablasser, 2022; Shae et al., 2019; Yang et al., 2022). Foremost, efforts center on promoting agonistic STING responses in the tumor microenvironment (TME), a strategy that has shown promising potential in multiple preclinical models against solid tumors and hematological malignancies (Chin et al., 2023; Guo and Huang, 2022; Li et al., 2021). STING agonists alone or combined with immune checkpoint blockade therapy are under clinical investigation for their potential as a new class of anticancer treatment (Meric-Bernstam et al., 2023). However, the clinical results of nucleotide-based cGAMP analogs, including ADU-S100 (MIW815) and MK-1454, have shown moderate and limited clinical responses in patients with advanced stage solid tumors or lymphomas (Meric-Bernstam et al., 2022; Samson and Ablasser, 2022). In addition to STING mutation, it is possible that interindividual differences in STING expression and functionality vary considerably between distinct tumors (Kitajima et al., 2022; Lee et al., 2022; Low et al., 2022). Moreover, cGAS has been implicated in tumor suppression independently of STING (Hu et al., 2021; Samson and Ablasser, 2022). Therefore, we next sought to apply the IDEA system for targeted delivery of the intracellular cGAS protein to boost antitumor immunity.

**Figure 2.**
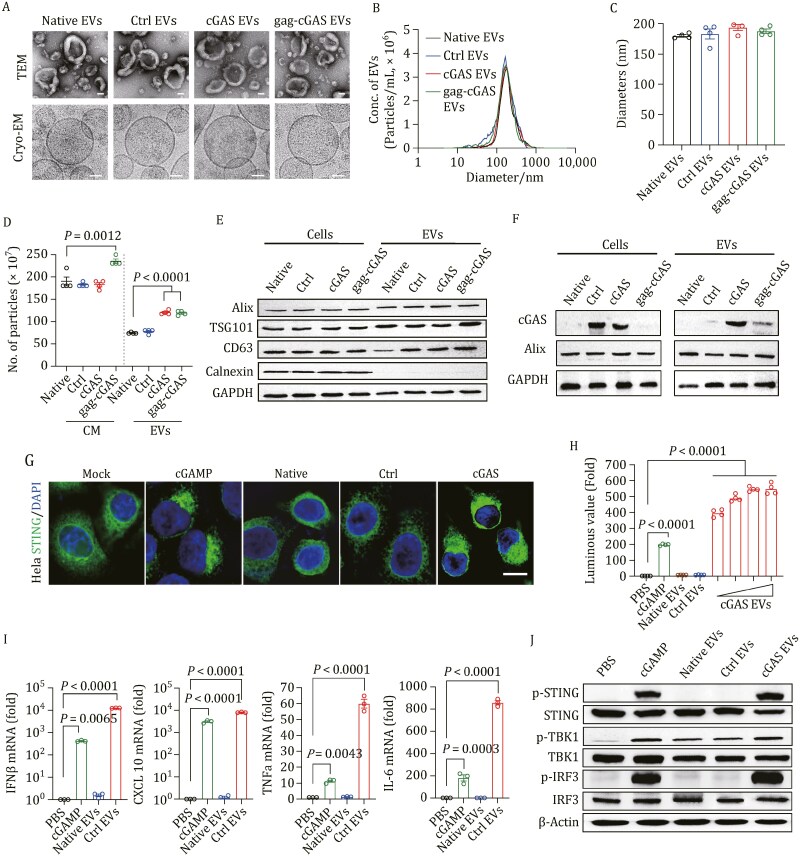
IDEA activates the STING signaling pathway through high-efficient intracellular delivery of cGAS. (A–F) Generation and characterization of various engineered EVs from producer cells. (A) Representative images of native EVs, Ctrl EVs, cGAS EVs and gag-cGAS EVs by transmission electron microscopy (TEM) and Cryo-TEM. Scale bar, 50 nm. (B–D) Nanoparticle tracking analyzer determined the size distribution (B), diameter (C) and total number of four types of purified EVs (D). (E) Western blot analysis of Alix, TSG101, CD63, and Calnexin in EVs and corresponding producer cells. (F) Western blot to determine the cGAS expression in producer cells and encapsulation in EVs. (G–J) High-efficient intracellular delivery of cGAS by IDEA activates the STING signaling pathway *in vitro*. (G) cGAS EVs, but not native EVs or Ctrl EVs, induce STING phase condensation after 4 h of incubation with 293T-STING-mNeonGreen cells. cGAMP was used as positive control. Scale bar, 10 μm. (H) cGAS EVs induce IFN luciferase in THP1-Lucia™ ISG cells. Data are presented as the mean ± SEM, *n* = 4 biologically independent experiments. (I) Relative *IFNβ, CXCL10, TNFα,* and *IL-6* mRNA levels show STING activation in THP-1 cells. Data are presented as the mean ± SEM, *n* = 3 biologically independent experiments. (J) THP-1 cells that treated with cGAS EVs exhibited STING activation and TBK-1/IRF-3 phosphorylation. All images in (A) and (G) are representative of at least three biologically independent experiments. Statistical analyses were performed using an ordinary one-way ANOVA with Tukey’s multiple comparisons test.

Highly purified EVs were generated after transient transfection of HEK293F cells with vectors encoding cGAS alone (“Ctrl EVs”), cGAS together with VSV-G (“cGAS EVs”) or gag-cGAS together with VSV-G (“gag-cGAS EVs”). Transmission electron microscopy (TEM) imaging displayed a “cup-shaped” morphology of all types of EVs (Fig. 2A). Cryogenic electron microscopy (Cryo-EM) of the isolated EVs also showed typical morphology (Fig. 2A). The size distribution and concentration of the EVs were further analyzed using nanoparticle tracking analysis (NTA), confirming the presence of EVs with a diameter in the range of 100–200 nm (Figs. 2B, 2C, S3A and S3B). Although particle yields were not majorly changed in conditioned medium, VSV-G co-transfection with either cGAS or gag-cGAS enhanced EV production (Fig. 2D). The presence of typical EV markers, such as CD63, Alix and TSG101, on EVs from producer cells was further confirmed by Western blot analysis (Fig. 2E). Purity of EVs was demonstrated by the absence of the endoplasmic reticulum-associated marker calnexin in EV lysates (Fig. 2E). Furthermore, we confirmed the stability of EVs after long-term storage (Figs. S3C–E). Finally, we confirmed that cGAS was highly expressed in engineered EVs from CAG::cGAS/VSV-G engineered producer cells (Fig. 2F). Collectively, these results point to the packaging of cGAS into EVs with high abundance via IDEA.

cGAS, as an intracellular DNA sensor, synthesizes cGAMP once it recognizes a diverse array of DNA substrates (Gao et al., 2013). cGAMP triggers the translocation of STING from the endoplasmic reticulum to the perinuclear region, where it forms puncta-like structures that are indications of STING oligomers (Wei et al., 2022). This change in STING subcellular localization was visualized in HeLa cells stably expressing GFP-tagged STING. There were no differences in the distribution or pattern of STING-GFP fluorescence observed between untreated cells and cells treated with native EVs or control EVs (Fig. 2G). In contrast, administration of cGAS EVs quickly induced significant STING translocation and puncta formation (Fig. 2G). To detect the role of STING activation, we utilized human THP1 monocyte (THP1-Lucia ISG) cells harboring a luciferase reporter gene under the control of a promoter comprising five IFN-stimulated response elements (ISRE) fused to an ISG54 minimal promoter. Compared to cGAMP, cGAS EV administration elicited substantially higher levels of ISRE reporter activity, which suggests that EVs entered cells and robustly released cargo (Fig. 2H). Furthermore, cGAS EVs significantly promoted the expression of downstream immunomodulatory cytokines in the STING pathway, including IFNβ, TNF-a and IL-6, and the chemokine CXCL10 (Fig. 2I), which are critical mediators of antitumor T-cell activation and recruitment. In addition, there was a noticeable increase in the levels of p-STING, p-TBK1, and p-IRF3 (Fig. 2J) following cGAS EV treatment. Similar responses were also confirmed in cells treated with cGAMP in the presence of digitonin. Taken together, these results demonstrated that EVs specifically deliver cGAS into the cytoplasm to activate STING signaling and robust interferon responses, enabling antitumor immunotherapy.

### Administration of EVs encapsulating cGAS shows therapeutic efficiency in multiple syngeneic tumor models

**Figure 3.**
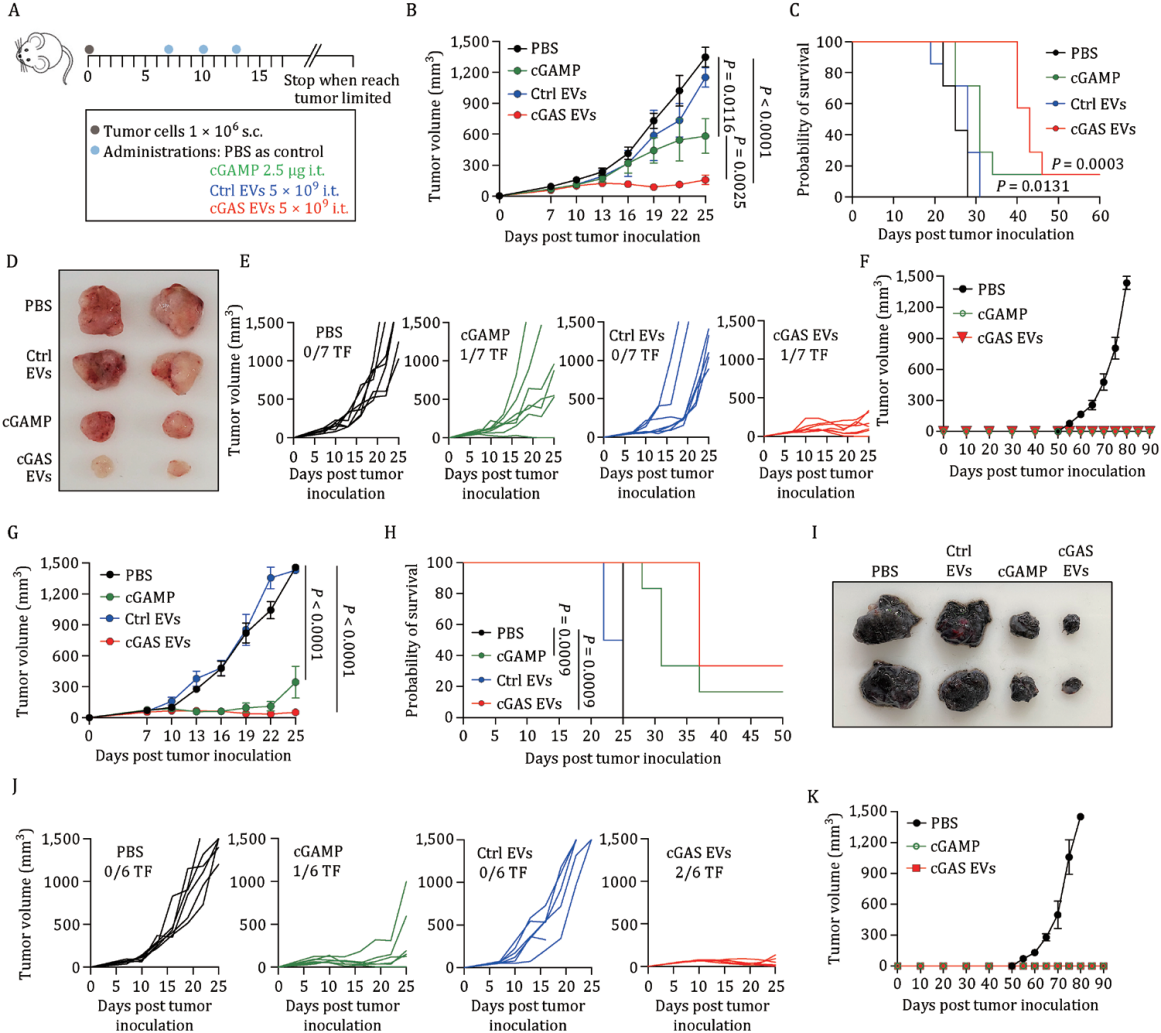
IDEA encapsulating cGAS shows therapeutic efficiency in multiple syngeneic tumor models. (A) Schematic of tumor inoculation, treatment schedule, and time points for analysis of tumor growth. MC38 (B–F) and B16.F10 (G–K) tumor-bearing mice were intratumorally administration of PBS vehicle, cGAMP (2.5 µg), Ctrl EVs (5 × 10^9^ particles) or cGAS EVs (5 × 10^9^ particles) at the indicated time points. Mean tumor volume (B and G), Kaplan-Meier survival curves (C and H), and spider plots of individual tumor growth curves (E and J) are shown. (D and I) Representative photographs of MC38 (D) and B16.F10 tumors (I) after treatments. (F and K) Mice showing complete responses (CR) to cGAS EVs and cGAMP treatment were rechallenged with tumor cells on the contralateral flank 50 days after CR without any further treatment. Data are mean ± SEM, *n* = 7 biologically independent mice in the MC38 tumor model and *n* = 6 biologically independent mice in B16.F10 tumor model. Statistical analyses among tumor volumes on day 25 in (B and G) were performed using ordinary one way ANOVA with Tukey’s multiple comparisons. Statistical analyses among survival curves in (C and H) were performed using a two-sided log-rank (Mantel-Cox) test.

Encouraged by the superior induction of STING signaling initiated by cGAS EVs *in vitro*, we then determined the therapeutic potential in tumor models. C57BL/6 mice were subcutaneously transplanted with MC38 murine colon adenocarcinoma cells, which are usually defined as “hot” tumors as they respond to immune checkpoint blockade (ICB) therapy (Fig. S4A–F). Once the tumors grew to 50–70 mm^3^, the mice experienced different treatments, including cGAS EVs, native EVs, free cGAMP, and vehicle [phosphate buffered saline (PBS)], through intratumoral injection three times (50 µL each time on Days 7, 10, and 13 after tumor cell inoculation), and the survival of the mice was monitored (Fig. 3A). We found that cGAS EV therapy dramatically decelerated tumor progression (Fig. 3B), and mice administered cGAS EVs survived significantly longer than PBS-treated mice, while control EVs did not provide a survival benefit (Fig. 3C). Similarly, tumor size and individual tumor growth curves confirmed that cGAS EVs exhibited the best antitumor efficacy (Fig. 3D and 3E). Treatment with cGAS EVs elicited a stronger response and a notable decrease in the tumor growth rate relative to cGAMP, although both treatments did not confer a significant survival difference. Remarkably, cGAS EV-treated mice rejected a rechallenge with MC38 cells on the opposite flank 50 days after recovery from original tumor inoculation, suggesting that cGAS EV administration induces a systemic antitumor response and the formation of antitumor immune memory (Fig. 3F).

To explore the universality of this strategy, we next evaluated the therapeutic efficacy of cGAS EVs in a poorly immunogenic B16.F10 melanoma cancer model (Fig. S4G–I). Similar to what was observed in the MC38 model, cGAS EV therapy delayed tumor growth (Fig. 3G) and resulted in a survival benefit with cures of ~40% of mice with no evidence of residual tumor two months posttreatment (Fig. 3H), suggesting that cGAS therapy has the potential to induce the desired antitumor response in nonresponsive cancer. Importantly, cGAS EV therapy eradicated one-third of established tumors compared with a milder response rate for cGAMP (Fig. 3I and 3J). We rechallenged these complete responders on the opposite flank and monitored the tumor volume. Without any additional treatment, all rechallenged mice completely resisted tumor growth for at least 90 days, whereas all the age-matched naive mice rapidly succumbed to their tumors (Fig. 3K). In addition, no direct growth inhibition was observed when tumor cells were treated with cGAS EVs *in vitro* (Fig. S5A–E), which implies that the antitumor effect may not result from the direct action of cGAS EVs on tumor cells. Meanwhile, none of the mice showed obvious body weight loss or damage to major organs during cGAS EV treatment, indicating that local delivery of cGAS EVs did not induce noticeable systemic adverse effects in mice (Fig. S6A–G). Taken together, these results prove that cGAS delivered by IDEA elicits robust antitumor immunity against multiple syngeneic tumor models and activates long-term antitumor immunity against tumor relapse. Our results document the high potential of IDEA as a biocompatible platform to improve the therapeutic efficiency of EV-based delivery systems targeting cGAS-STING signaling.

### cGAS EVs shift the immunocellular composition of the TME and elicit enhanced antitumor immunity when synergized with ICB

**Figure 4.**
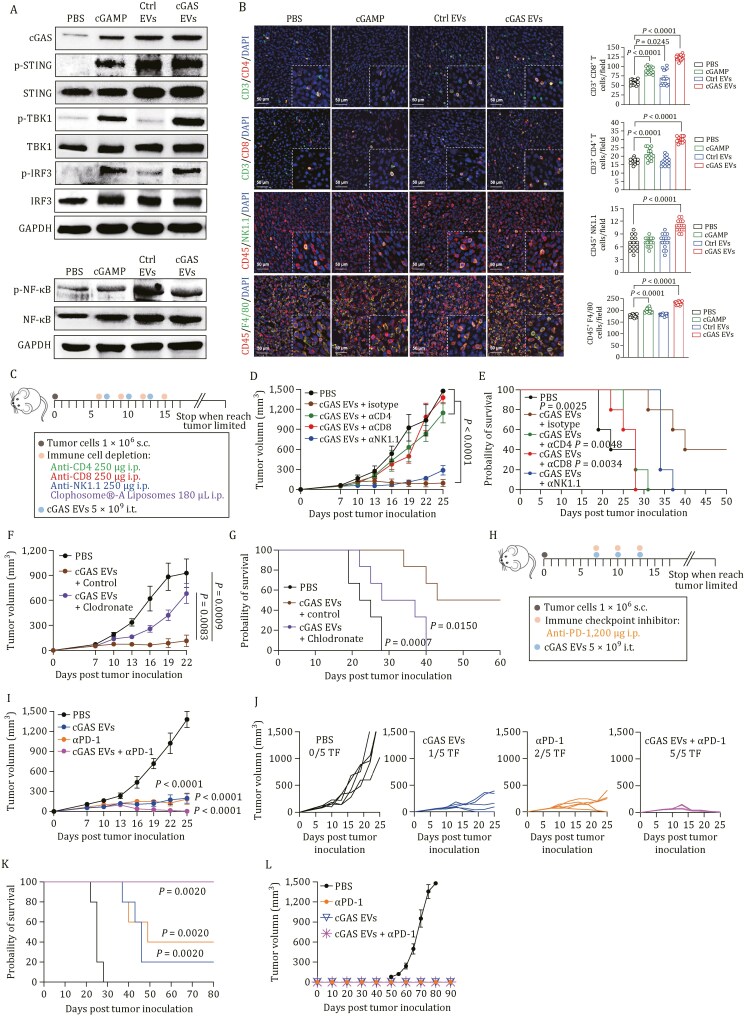
IDEA activates STING signaling and the immune cell response in colon cancer ***in vivo***. (A) MC38 tumor tissues were collected 4 h after the second dosage of treatment. Western blot analysis of the cGAS-STING signaling pathway showed that mice treated with cGAS EVs display an abundant expression of phosphorylated TBK-1/IRF-3. (B) Representative fluorescence images of infiltrated immune cells in tumor after different treatments accompanied by statistical analysis of the number of infiltrated immune cells. (C) Schematic of tumor inoculation, treatment schedule, and time points for analysis of tumor growth. (D–G) Tumor growth of MC38 tumor-bearing C57BL/6 mice treated with cGAS EVs only (*n* = 5) or along with depletion of NK cells (*n* = 5), CD4^+^ T cells (*n* = 5), CD8^+^ T cells (*n* = 5) and macrophages (*n* = 6) at the indicated time points. (H–K) C57BL/6 mice were s.c. inoculated with 1 × 10^6^ MC38 tumor cells in the right flank and then intratumorally administrated with PBS vehicle (*n* = 5), cGAS EVs (5 × 10^9^ particles, *n* = 5), intraperitoneal injection of anti-PD-1 antibody (200 µg, *n* = 5) or combined treatment (cGAS EVs + αPD-1, *n* = 5) at the indicated time points. Mean tumor volume (D, F, and I), Kaplan-Meier survival curves (E, G, and K) and spider plots of individual tumor growth curves (J) are shown. (L) Mice showing complete responses (CR) to cGAS EVs, anti-PD-1 antibody or combined treatment (cGAS EVs + αPD-1) were rechallenged with tumor cells on the contralateral flank 50 days after CR without any further treatment. All images in (B) are representative of at least three biologically independent experiments. Statistical analyses in (B) were performed using ordinary one way ANOVA. Statistical analyses in tumor volume in (D, F, and I) were performed using ordinary one way ANOVA with Tukey’s multiple comparisons, respectively. Statistical analyses among survival curves in E, G, and K were performed using a two-sided log-rank (Mantel-Cox) test, respectively. Data are presented as the mean ± SEM.

We performed a pathological analysis of tumor tissues to verify the therapeutic effect after different treatments as indicated above. Accordingly, the proteins involved in the cGAS-STING signaling pathway were dramatically upregulated by cGAS EVs, as detected by western blot analyses (Fig. 4A), indicating the great promise of EV-based cGAS delivery to induce STING pathway activation for therapeutic applications *in vivo.* Having proven the impressive antitumor effect, we first examined the effects of cGAS EVs on tumor cells, and was a difference observed with cGAS delivery on cell apoptosis and interferon signaling (Fig. S5A–E). Subsequently, we explored the corresponding immune response in the tumor model via immunofluorescence staining. First, the dominant immune cell subsets in the tumor tissue were analyzed from MC38 tumor-bearing mice. Obviously, cGAS EV injection elevated the levels of CD3^+^CD8^+^ (cytotoxic) and CD3^+^CD4^+^ (helper) T cells in tumor tissues (Fig. 4B). Meanwhile, the percentage of CD8^+^ T cells was the highest in the cGAS EV-treated group, thus potentiating the adaptive immune response for tumor control. Additionally, cGAS EVs significantly increased the number of infiltrating NK1.1^+^ NK cells and F4/80^+^ macrophages (Fig. 4B). To further investigate the immune cell-dependent contribution to the robust therapeutic efficacy of cGAS EVs, CD4^+^ T cells, CD8^+^ T cells, NK cells, and macrophages were depleted using appropriate antibodies or clodronate (Figs. 4C and S7A–D). Depletion of NK cells showed minimal antitumor effects, whereas the depletion of CD4^+^ or CD8^+^ T cells resulted in a significant decrease in both tumor growth inhibition and survival benefit (Fig. 4D and 4E), suggesting that T cells play a critical role in cGAS-initiated immunotherapy. Furthermore, macrophage depletion also led to a failure of therapy, implying the essential role of macrophages in the antitumor effects of cGAS (Figs. 4F, 4G, and S5F). Collectively, these data suggest that tumor regression after cGAS EV therapy correlated with both the innate and adaptive immune systems.

Immune checkpoint blockade, specifically targeting the PD-1/PD-L1 axis, mitigates T-cell exhaustion but is only effective in a subset of patients with cancer (Morad et al., 2021). Furthermore, individuals treated with STING agonists showed increased expression of PD-1 in tumor-infiltrating lymphocytes (TILs) and PD-L1 in TME cells (Meric-Bernstam et al., 2022, 2023). Therefore, we further investigated whether cGAS delivered by IDEA could induce susceptibility to anti-PD-1 therapy in syngeneic mouse tumor models. Accordingly, we hypothesized that EVs loaded with cGAS, by activating both innate and adaptive immune cells, have the potential to induce the desired antitumor response. Subsequently, the “EV-primed” tumor would be more sensitized to anti-PD-1 treatment, resulting in an effective efficacy. In the MC38 model, the combination of anti-PD-1 with cGAS EVs showed a strong synergistic antitumor effect compared with either treatment alone (Fig. 4H), leading to complete tumor rejection in all mice; 100% of mice remained tumor-free after 80 days (Fig. 4I–K). More excitingly, mice cured by cGAS and ICB exhibited resistance to tumor rechallenge, indicating that this combination therapy could elicit effective and long-lasting immune memory that protected the mice from tumor relapse (Fig. 4L). Collectively, these results confirm the synergistic effect of cGAS EVs and ICB, offering an opportunity to utilize cGAS EVs delivered by IDEA in patients with cancers that only partially respond to ICB.

### Single-cell analyses delineate TME remodeling following cGAS therapy

**Figure 5.**
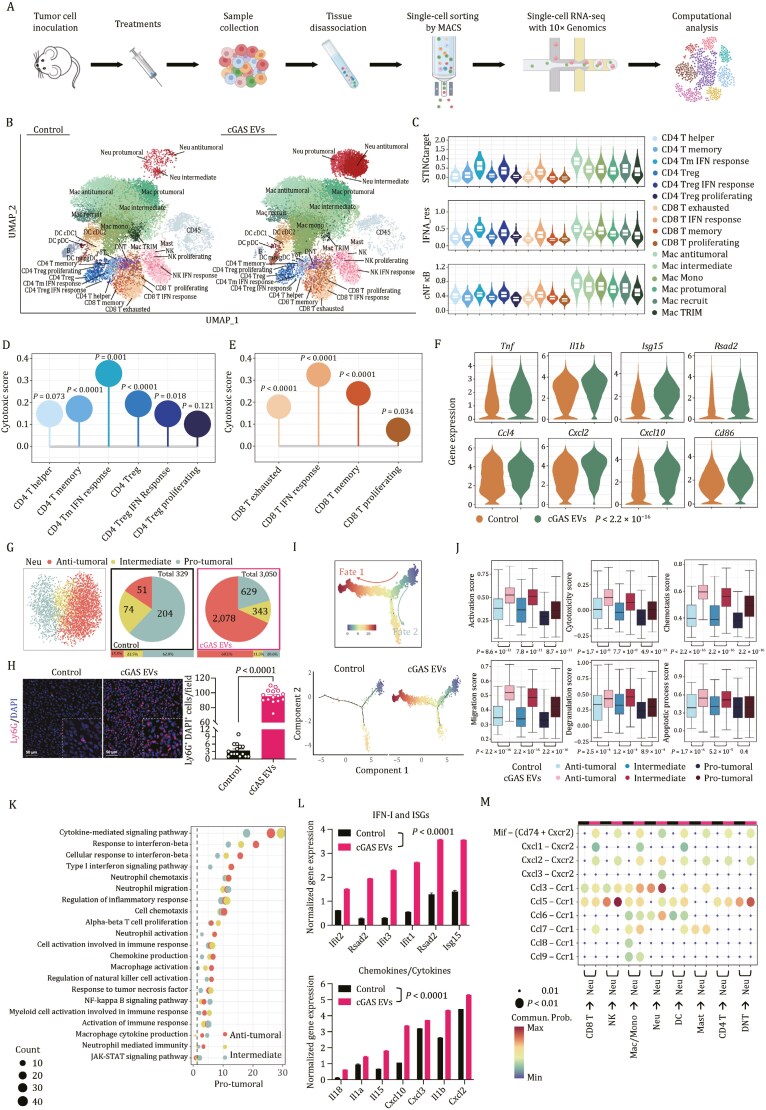
Single-cell analyses delineate myeloid and lymphoid compartment remodeling following cGAS delivery by IDEA. (A) Schematic diagram of the scRNA-seq experimental design. (B) Uniform manifold approximation and projection (UMAP) projection of all cells after QC and filtering (as in Fig. S8A), with each cell type represented in a different color. (C) Violin plots showing the gene signature scores of canonical STING targets, IFNα response genes and NF-κB target genes in CD4^+^ T cells, CD8^+^ T cells and macrophages. (D and E) Cytotoxic score for each cell subtype of CD4^+^ T (D) and CD8^+^ T (E), respectively. (F) Violin plots comparing the expression levels of selected antitumoral genes in macrophages between the two groups. (G) UMAP illustration of scRNA-seq data of neutrophils from PBS- and cGAS EV- treated mice, colored by cell subpopulations. The pie chart depicts the number and ratio of neutrophil subpopulations in each group. (H) Representative immunofluorescence images of Ly6G^+^ neutrophils counterstained with DAPI in the PBS and cGAS EV groups. Quantification of Ly6G^+^ cells per microscopic field by manual counting. (I) Pseudotime ordering of neutrophils into a major trajectory with two bifurcations which are indicated with arrows (upper panel), and distribution of the pseudotime trajectory of the PBS and cGAS EV groups (lower panel), colored by inferred pseudotime ordering. (J) Boxplots comparing the neutrophil-associated signature scores in each subpopulation of the two groups. (K) Scatter plot representing the terms enriched by GO and KEGG enrichment analysis based on the up-regulated differentially expressed genes in the cGAS EV group (versus the PBS group) with dot color and size representing the cell type and number of genes in the corresponding terms respectively. The dashed line indicates −log_10_(*P*.adj) equal to 0.05. (L) Summary of selected differentially expressed genes in the PBS and cGAS EV groups. (M) Bubble heatmap showing predicted ligand–receptor interactions between neutrophils and other immune cell types with dot color and size representing the *P* value and probability of communication, respectively. Statistical analyses in (D, E, H, and L) were performed using a two-sided unpaired *t*-test. Statistical analyses in (F and J) were performed using two-sided unpaired Wilcoxon test.

To elucidate the complexity of cellular compositions in tumors and determine whether cGAS EV therapy constrained tumor progression by promoting a cGAS-mediated antitumor immune response, we isolated CD45^+^ tumor-infiltrating immune cells on day 10 post-implantation of MC38 colon cancer cells and performed single-cell RNA sequencing (scRNA-seq) (Fig. 5A). After quality control and filtering of potential doublets (Fig. S8A), we obtained 49,741 high-quality single cells in total (26,880 from cGAS therapy and 22,861 from control) and cataloged them into eight major cell lineages, including T cells, B cells, natural killer (NK) cells, and diverse myeloid-lineage cells in both groups, annotated by canonical marker genes (Fig. S8B–G). Further analysis of scRNA-seq data showed that IFN response-specific populations (Fig. 5B) expressed high levels of canonical STING targets, IFNα response genes and NF-κB target genes (Fig. 5C). Gene Ontology (GO) and Kyoto Encyclopedia of Genes and Genomes (KEGG) enrichment analysis showed that these IFN response populations were enriched in myeloid chemotaxis and cytokine/chemokine-mediated signaling pathways (Fig. S8H).

Given that antibody-mediated depletion of CD4 and CD8 T cells abolished the antitumor efficacy of cGAS EVs, whereas blockade of NK cells exhibited minimal effects, we first focused on the intrinsic properties and potential functions of T-cell populations in tumors. By unsupervised clustering, we identified six CD4^+^ clusters (Fig. S9A–D). GO/KEGG enrichment analysis revealed that immune-associated pathways (such as regulation of interferon production and adaptive immune response and pattern recognition receptor signaling pathway) were enriched in IFN response CD4 subpopulations (Fig. S9E). For CD8^+^ T cells, we noticed that all CD8^+^ T cells expressed increased levels of genes encoding activation and effector molecules (such as *Ifng*, *Gzma*, *Gzmb,* and *Nkg7*) (Fig. S9F) in the cGAS group compared with the control, indicating the intrinsic antitumor potential of T-effector cells inside tumors. We then categorized CD8^+^ T cells into four clusters and found that the proportions of the CD8 T-cell IFN response increased, whereas the number of exhausted CD8 T-cell subsets decreased (Fig. S9G–I). GO/KEGG enrichment analysis revealed that CD8 T IFN response subpopulations displayed upregulation in pathways associated with the regulation of leukocyte-mediated cytotoxicity and NF-κB/TNF signaling (Fig. S9J). Relative to the control group, cGAS therapy induced significant upregulation of genes involved in the defense response to virus, cell killing, and T-cell-mediated immunity in the CD8 T IFN response subset (Fig. S9K). Consistent with these results, cGAS therapy led to a significant upregulation of cytotoxic genes in both CD4^+^ and CD8^+^ subtypes (Fig. 5D and 5E). These findings indicate that cGAS EVs change the intratumoral T-cell population from one showing characteristics of exhaustion/dysfunction to one showing signs of reactivation.

The most abundant population in the TME of these tumors was monocytes/macrophages, which represented more than 70% of CD45^+^ cells. Therefore, we looked more closely at the subpopulation composition defined by scRNA-seq. Six major monocyte/macrophage subpopulations were identified by unbiased clustering (Fig. S10A–D). Monocytes/macrophages from progressively growing tumors in control mice expressed high levels of *Tgfb1*, *Ctsa,* and *Cxcr4*, markers frequently associated with anti-inflammatory and immunosuppressive microenvironments (Fig. S10E). On the contrary, cGAS induced higher expression levels of *Tnf*, *Il1b,* and *Cxcl10*, etc., which are markers associated with a classical IFN-activated, proinflammatory state that represents increased antitumor ability (Fig. 5F). Consistent with this hypothesis, GO/KEGG enrichment analysis also suggested that cGAS EV treatment increased antitumor macrophage function, with pathway enrichment in the regulation of the innate immune response, neutrophil migration, NF-κB/TNF signaling, and chemokine-mediated signaling (Fig. S10F), implying that cGAS EVs shift protumoral macrophages into an antitumoral state. Moreover, immunostaining of iNOS and CD206 on tumor sections showed the percentage of iNOS^+^ M1 macrophages increased and CD206^+^ M2 macrophages significantly decreased (Fig. S10G and S10H). Based on these data, we conclude that cGAS delivery converts macrophages from an immunosuppressive state to an immunogenic, tumoricidal state, emphasizing the immunoregulatory role of cGAS in the antitumor immune milieu.

To our surprise, the most noticeable difference in the TME in mice receiving cGAS EVs was a tremendous increase in neutrophils compared to control animals (Fig. 5B and 5G). These observations were further supported independently by immunofluorescence staining: administration of cGAS EVs led to a dramatic increase in tumor-infiltrating Ly6G^+^ neutrophils (Fig. 5H). A growing number of studies support the potential for neutrophils to perform antitumor functions (Cui et al., 2021; Gungabeesoon et al., 2023; Ponzetta et al., 2019), although neutrophils are often initially co-opted by cancers to promote immunosuppression, tumor growth, and metastasis (Wang et al., 2023a, 2023b). The apparently contradictory roles of neutrophils in cancer are likely the result of differences in the tumor microenvironment affecting neutrophil maturation, activation, and functional states (Hedrick and Malanchi, 2022; Quail et al., 2022). Nonetheless, increasing evidence demonstrates that manipulation of the tumor milieu can result in the infiltration and activation of tumor-killing neutrophils that drive T-cell-independent tumor clearance (Hirschhorn et al., 2023; Linde et al., 2023). Hence, we sought to gain deeper insight into the infiltrating neutrophil subpopulations and decipher how such changes might correlate with or affect successful antitumor responses.

Do neutrophil subsets differ in the expression of genes associated with pro- and antitumoral activity, or does EV therapy modulate such genes within the cell subsets? By unsupervised clustering, neutrophils infiltrating the TME exhibited three different phenotypes (Figs. 5G and S11A). Notably, cGAS therapy dramatically augmented the number of antitumoral neutrophils. By pseudotime analysis, the protumoral subset was mainly distributed along fate 2, while the antitumoral subset aggregated in fate 1 (Fig. S11B). Pseudotime ordering showed that cGAS therapy enhanced the differentiation of neutrophils into fate 1 cells with the potential for antitumor immunity (Fig. 5I). Furthermore, genes upregulated in fate 1 and downregulated in fate 2 were involved in pathways including antigen processing and presentation, leukocyte-mediated cytotoxicity and chemotaxis, neutrophil degranulation, phagocytosis, and cell killing (Fig. S11C and S11D). Neutrophil-associated signature scores (Figs. 5J and S11E) in three neutrophil subpopulations between the two groups also indicated enhanced antitumor functionality of infiltrating neutrophils following cGAS EV administration. We further characterized the gene expression profiles in neutrophils and found that cGAS EVs triggered significant upregulation of IFN regulatory factors, ISGs, proinflammatory cytokines, and leukocyte-recruiting chemokines, consistent with the specific enrichment of pathways involved in cytokine-mediated signaling, the interferon response and activation of the immune response (Fig. 5K and 5L). When we studied the interaction between neutrophils and other immune cells, the data revealed an increasing number of communications after cGAS administration, especially between neutrophils and macrophages (Figs. 5M, S11F and S11G). There was a much stronger chemotaxis-related interaction among antitumoral/intermediate neutrophils, whereas the communication in the protumoral subset decreased (Fig. S11G), which suggests that cGAS EVs triggered a multifaceted shift to an inflamed and tumoricidal microenvironment. To confirm the biological functions of neutrophils in the cGAS EV-mediated antitumor effects, we depleted the neutrophils through anti-Ly6G treatments post-tumor initiation. We observed that neutrophil depletion led to a failure of therapy, implying the essential role of neutrophils in the antitumor effects of cGAS EVs (Fig. S12A–E). Collectively, our data suggest the function of neutrophils in enhancing antitumor immunity and reshaping the immunosuppressive tumor environment.

In summary, we delineated a comprehensive landscape of the TME via scRNA-seq by comparing the unique cellular compositions after cGAS EV administration. Unbiased assessment of the gene expression of tumor-infiltrating cells revealed significant remodeling of both the intratumoral lymphoid and myeloid compartments. Our findings highlight the role of cGAS delivered by IDEA, which leads to suppression of tumor progression, accompanied by enhanced antitumor immune responses and amelioration of the immunosuppressive tumor microenvironment through the enforced activation of immune cells in tumor-bearing mice.

## Discussion

The development and clinical use of protein drugs such as immunoglobulins and cytokines, both of which target extracellular or cell surface molecules, have resulted in a powerful new class of therapeutics (Muttenthaler et al., 2021). On the basis of intracellular targets, the rational design of efficient cytosolic or nuclear delivery carriers holds enormous promise for biotherapeutic development (Goswami et al., 2020; Tian et al., 2022). EVs, as natural nanoparticles, benefit from favorable safety profiles and unique biodistribution capabilities, rendering them attractive drug delivery modalities over synthetic analogs (Kalluri and LeBleu, 2020; Wiklander et al., 2019) (Wang et al., 2022). Generally, the previous widely used approaches require co-expression of the desired protein with viral capsid proteins such as Gag (Votteler et al., 2016) or direct fusion of target proteins to EV biogenesis-related scaffold proteins, such as ARRDC1, PTGFRN, and LAMP2A/B, etc. (Dooley et al., 2021; Ferreira et al., 2022; Silva et al., 2021; Wang et al., 2018; Zheng et al., 2023), which may interfere with the function of the protein of interest. In this study, we developed an engineered EV platform, “IDEA,” which is highly modulatory, versatile, and robustly delivers multiple bioactive proteins for therapeutically relevant *in vitro* and *in vivo* applications. In short, by addressing the distinct bottlenecks of EV-mediated delivery, our IDEA platform incorporated higher levels of intracellular proteins within EVs and fulfilled efficient cargo release after uptake.

When compared with virus-based payload delivery, EV-based systems have the advantage of being less immunogenic and easier to manufacture (Greening et al., 2023; Herrmann et al., 2021). Besides, IDEA (i) allows a rapid onset of intracellular biological activity (e.g., Cre enzyme is delivered in its active form and induces recombination rapidly after entering a cell, while AAV or lentivirus require a time delay for Cre recombinase to be expressed from the Cre gene); (ii) delivers cargo directly and transiently into recipient cells, and in the context of transcription factors or gene-editing tools, IDEA can potentially avoid unwanted off-target and knock-on effects caused by prolonged expression; and (iii) does not involve potential risks associated with DNA integration within the host genome. Another intriguing characteristic of EVs is the capability to concurrently encapsulate multiple cargos, including the surface display of targeting entities for cell or tissue specificity. Combined, due to its modularity, flexibility, and versatility, IDEA technology could potentially be used to generate EVs delivering a wide range of functional proteins for therapeutic applications. It should be noted, however, that the protocols described here are not meant to supplant AAV/lentivirus or LNP technology but rather to complement them.

Therapeutic targeting of the cGAS-STING pathway remains a substantial challenge: neither the natural ligands cyclic dinucleotides (CDNs) nor small-molecule STING agonist compounds have shown remarkable efficacy in preclinical studies (Meric-Bernstam et al., 2022, 2023). The efficient delivery of cGAS, as the upstream modulator of STING signaling, would be a novel promising approach for antitumor therapy. Encouragingly, our results demonstrated that administration of cGAS EVs shows excellent efficacy in controlling tumor growth across multiple murine cancer models, eliciting systemic antitumor immunity that can protect against tumor rechallenge. The engineered EVs enhance the intracellular delivery of cGAS via an endosomal escape mechanism, preferentially activating STING in myeloid cell populations within the TME to trigger a multifaceted shift to a “hot” T-cell-inflamed TME that inhibits tumor growth. Owing to both their membrane-penetrating properties and immune-stimulatory activity, cGAS EVs provide advantages over existing STING agonists. Systemic administration of immunotherapies can cause off-target side effects, such as inflammation or autoimmunity (Liu et al., 2019). However, local injection constitutes a feasible strategy to reduce the risk of systemic toxicities and achieve higher local bioactive drug concentrations. Regarding the mechanism of cGAS EVs in antitumor immunity, we do not rule out the possibility that other mechanisms of cGAS-mediated immunotherapy may be involved, especially in the heterogeneous TME. In addition, while not explored herein, an attractive feature of cGAS EVs is the ability to efficiently encapsulate a diversity of cargos, offering opportunities for co-delivery with other intracellular immunomodulators.

Currently, VSV-G co-expression in EVs is critical to our IDEA strategy for functional delivery of intracellular proteins, although we have not determined the underlying molecular mechanisms. Similarly, VSV-G is also used in the latest studies of eVLPs for efficient delivery of nuclear proteins such as CRISPR base-editors (Banskota et al., 2022). Since VSV-G is a viral envelope protein, it should be further tested to determine whether its immunogenicity may be detrimental, especially for applications beyond boosting immune response in cancer immunotherapy as we described in this study. Moreover, improving EV targeting/specificity is also an urgent problem to be solved. In particular, we envision that payloads targeted for endocytosis by specific cell surface receptors could be obtained through membrane engineering on EV-producing cells. Thirdly, further experiments are clearly required to determine whether the intracellular delivery of the cGAS downstream molecules, such as STING, TBK1, or IRF3, could elicit better efficacy. Finally, since our study involves cancer immunotherapy studies performed in mice, future studies will need to evaluate whether cGAS EVs also elicit more potent and broad antitumor responses in nonhuman primates and humans. In summary, we present a novel technology to efficiently generate engineered EVs for the intracellular transfer of bioactive proteins. The facile IDEA platform is versatile and could also be applied for the delivery of other intracellular proteins, such as CRISPR enzymes or other therapeutic proteins, which would have a significant impact on medicine.

## Materials and methods

### Cell culture

Mouse colon cancer cells MC38, melanoma cells B16.F10, and human monocyte THP-1 cells were originally purchased from Procell Life Science & Technology Co. Ltd. HeLa, HEK293T, and HEK293F cells were originally purchased from the American Type Culture Collection. All cell lines were free from mycoplasma and authenticated with short tandem repeat (STR) profiling using RT-qPCR. MC38, B16.F10, HeLa, and THP-1 cells were cultured in Dulbecco’s modified Eagle’s medium (DMEM) and RPMI 1640 supplemented with 10% fetal bovine serum. HEK293F cells were cultured in the Union-293 (Union-Biotech) medium under continuous shaking at 175 rpm. All cells were cultured in a humidified incubator containing 5% CO_2_ at 37°C.

### Animals

Female C57BL/6 mice (aged 6–8 weeks) were obtained from GemPharmatech Co. Ltd. Animals were maintained at the animal facilities under specific-pathogen-free conditions under a 12 h–12 h light-dark cycle. All animal studies were performed according to protocols approved by the Ethics Committee at the University of Science and Technology of China (USTCACUC24110123017). Mice were allowed to acclimate to the experimental housing facility for at least 3 days before tumor injections.

### Construction of vectors

For the VLP system, a coding sequence of Cre was inserted to replace the corresponding Cas9 fragment in pCMV-MMLVgag-3xNES-Cas9 (Addgene #181752) through In-Fusion cloning (Vazyme). For the EV system, the CAG promoter in pCAG-Cre (Addgene #13775) was replaced with CMV, EF1a, or PGK to create pCMV-Cre, pEF1a-Cre, and pPGK-Cre vectors. Codon-optimized DNA sequences coding for β-catenin and cGAS were PCR amplified and cloned downstream of the CAG promoter in pCAG-Cre. All expression cassettes were confirmed by SANGER sequencing.

### Isolation and purification of EVs

HEK293F cells were transfected with the corresponding constructs by PEI transfection reagent (Yeasen). Conditioned medium (CM) was harvested for EV isolation. Briefly, CM was collected by centrifugation at increasing speeds: 300 ×*g* for 10 min and 2,000 ×*g* for 10 min. Then, the cell-free, debris-free CM was centrifuged at 120,000 ×*g* for 90 min at 4°C, followed by two washes with PBS using a benchtop ultracentrifuge with an MLA-50 rotor (Beckman Coulter, Optima MAX-XP). Further purification of EVs was conducted according to the manufacturer’s protocol (IZON) as described previously (Han et al., 2021). All EVs were used directly or stored at −80°C.

### Nanoparticle tracking analysis (NTA)

As described previously (Liu et al., 2019), the particle concentration and size distribution of EVs were quantified using a nanoparticle tracking analyzer (Particle Metrix, ZetaView PMX110) equipped with ZetaView 8.04.02 SP2 software according to the manufacturer’s protocol.

### Cryo/Transmission electron microscopy

For TEM analysis, EVs were diluted to 1.5 × 10^11^/mL and incubated for 1.5 min on a 200-mesh Formvar^TM^ and carbon-coated copper grid (Ted Pella, 1GC300). The grids were rinsed with water and stained with 2% uranyl acetate solution. Then, the grids were allowed to dry prior to imaging with a transmission electron microscope (FEI, Tecnai F12) operating at 80 kV at the University of Science and Technology of China. For Cryo-TEM, EVs were applied on a glow-discharged 300 mesh EM grid with lacey carbon (Ted Pella, 01883-F) and were vitrified using a Vitrobot (FEI, Mark IV). Grids were mounted in a Gatan 626 cryo-holder, and two-dimensional automated data were obtained by cryo-transmission electron microscopy (FEI, Tecnai G2 F20) operated at 200 kV at the University of Science and Technology of China.

### Luciferase reporter assay

THP1-Lucia™ ISG cells express the secreted luciferase (Lucia) reporter gene under the control of five interferon-stimulated response elements (ISRE). This ISRE luciferase activity of Lucia ISG cells represents the activation of STING signaling. Cells were stimulated with cGAMP, native EVs, Ctrl EVs, or increasing concentrations of cGAS EVs for 13 h. The ISRE reporter activity was determined following the standard protocol (Invitrogen).

### STING-GFP trafficking assay

HeLa cells stably expressing the STING-GFP gene were cultured on glass coverslips overnight. The cells were then stimulated with cGAMP, native EVs, Ctrl EVs, and cGAS EVs for 4 h. After treatment, cells were fixed and imaged with fluorescence microscopy (Leica, DMi8).

### RT-qPCR

Total RNA was isolated using a FastPure Cell/Tissue Total RNA Isolation Kit (Vazyme) according to the manufacturer’s instructions. After reverse transcription using a reverse transcription reagent kit (Takara) and oligo (dT) primers, real-time quantitative PCR was performed using SYBR Green PCR Mix (Takara) and a LightCycler detection system (Roche, Synergy H1) as described (Liu et al., 2021). The sequences of primers used in this study were as follows: human *IFNβ*: AGGACAGGATGAACTTTGAC, TGATAGACATTAGCCAGGAG; human *TNFα*: TTCTCCTTCCTGATCGTGGC, ATGATCTGACTGCCTGGGCCAG; human *IL-6*: AGACAGCCACTCACCTCT TCAG, TTCTGCCAGTGCCTCTTTGCTG; human *CXCL10*: GCCGTCATTT TCTGCCTCA, CGTCCTTGCGAGAGGGATC; and human *GAPDH*: ATGACATC AAGAAGGTGGTG, CATACCAGGAAATGAGCTTG. Relative gene expression was normalized to *GAPDH*.

### Western blot analysis

Total protein from cells, EVs, and tissues was extracted using RIPA lysis buffer containing protease and phosphatase inhibitors (TargetMol). Protein concentration was quantified by BCA protein assay (Biosharp) according to the manufacturer’s recommendations. SDS-PAGE was performed with 30 μg of total protein using a 12% tris-glycine gel. Proteins were detected and quantified using the SH-Compact523 gel imaging system (Shenhua Science Technology) with HRP-linked secondary antibodies (Proteintech) and normalized to GAPDH or β-actin. Primary antibodies used for this study were purchased from Cell Signaling Technology unless otherwise stated: Cre (Meck-Millipore, 69050-3), β-catenin (8480S), Alix (2171S), CD63 (Santa Cruz, sc-5275), TSG101 (Servicebio, GB11618), Calnexin (2433S), cGAS (D1D3G), p-STING (Thermo Fisher, PA5-105674), STING (13647S), TBK1(3504S), p-TBK1 (5483S), p-IRF3 (29047S), NF-κB (8242S), IRF3 (Biolegend, 655701), p-NF-κB (Servicebio, GB113882), β-actin (Proteintech, 66009-1-Ig), and GAPDH (Proteintech, 10494-1-AP).

### Flow cytometric analysis

For quantification of Cre delivery, 293T reporter cells were collected for GFP analysis after incubation with EVs for 48 h. For quantification of cell viability, MC38 cells were treated with cGAMP, Ctrl EVs, cGAS EVs, and gag-cGAS EVs for 24 h. The cells were then suspended and stained with Fixable Viability Dye according to the protocol supplied by the manufacturer (Invitrogen). The fluorescence signal of the cells was acquired and analyzed using flow cytometry (BD, LSRFortessa).

### *In vivo* studies

MC38 and B16.F10 tumors were grown by subcutaneous (sc) implantation of 5 × 10^5^ or 1 × 10^6^ cells in 50 µL of PBS in the right flank (of shaved mice). When the tumor volume reached ~50–70 mm^3^, tumor-bearing mice were randomly assigned and intratumorally injected with PBS, cGAMP, Ctrl EVs, or cGAS EVs, as appropriate. Tumor size and body weight were measured and recorded every 3 days. Tumor volume was calculated with the formula (length × width^2^)/2. Animals were euthanized if tumors developed open ulcerations or reached volumes > 1,500 mm^3^ or when body weight loss exceeded 20%. For *in vivo* synergistic immunotherapy, mice were intraperitoneally (i.p.) injected with 250 μg checkpoint inhibitor anti-PD-1 antibody (Bioxcell, BE0146) or cGAS EVs for a total of three doses. For the rechallenge studies, mice with long-term survival from specific groups were inoculated with 1 × 10^6^ MC38 cells or 1 × 10^6^ B16.F10 cells on the flank opposite the initial tumor injection.

### Immunofluorescence staining

Tumors were collected from mice, washed with 1× PBS twice, fixed in periodate-lysine-paraformaldehyde for 16–24 h, dehydrated in 30% sucrose for 24 h, and then snap frozen in OCT. Tumor tissue in OCT was sectioned at 10 μm thickness, blocked for 60 min in staining buffer (0.1 mol/L Tris, 1% BSA, 1% FBS, 0.3% Triton X-100), and stained with fluorescently conjugated or unconjugated antibodies overnight at 4°C. Slides were washed with 1× PBS and further stained for 1 h with fluorescently conjugated secondary antibodies for unconjugated antibodies following the protocol (Ma et al., 2019). Antibodies against CD45 (GB113886), CD4 (GB15064), CD8 (GB114196), F4/80 (GB113373), Ly6G (GB11229), iNOS (GB11119), and CD206 (GB113497) were purchased from Servicebio. Antibodies against NK1.1 (39197) were purchased from Cell Signaling Technology. Antibodies against tdTomato/RFP (600-401-379) were purchased from ROCKLAND. Immunofluorescence images were taken under fluorescence microscopy (Leica, DMi8) and analyzed using LAS X Navigator software.

### Safety studies

Once reaching the tumor size endpoint, blood was harvested, allowed to clot, and used to prepare serum for analysis. The serum was tested by a chemistry analyser (Semens, Advia 2400) for levels of albumin (ALB), alkaline phosphatase (ALP), aspartate aminotransferase (AST), cholesterol (CHOL), direct bilirubin 2 (DBIL-2), high-density lipoprotein (HDL), low-density lipoprotein (LDL), enzymatic creatinine 2 (ECRE-2), glutathione (GLUH3), lipoprotein particles (LDLP), total bile acid (TBA), and triglycerides (TRIG-2). Selected organs were harvested for analysis of toxicity. Frozen sections of selected organs were subjected to H&E staining using the same method as reported previously (Ma et al., 2019).

### Depletion of immune cells *in vivo*

As previously described (Wang et al., 2020), to deplete immune cells *in vivo*, tumor-bearing mice were intraperitoneally injected with anti-CD4 (Bioxcell, BE0003), anti-CD8α (Bioxcell, BE0061), anti-NK1.1 (Bioxcell, BE0036), anti-Ly6G (Bioxell, BE0075), or isotype control (BioXcell, BE0090, BE0085) antibodies at an initial dose of 250 μg 1 day before treatment, followed by 250 μg every 3 days. To deplete macrophages, tumor-bearing mice were intravenously injected with 180 μL Clophosome®-A liposomes (FormuMax, F70101C) at an initial dose of 180 μL 1 day before treatment, followed by 100 μL every 3 days. Plain control liposomes (FormuMax, F70101) were used as controls. Depletion of CD4^+^ T cells, CD8^+^ T cells, NK cells, macrophages, and neutrophils was confirmed using flow cytometry analysis.

### Tumor dissociation

Tumor samples were harvested 4 h after the second dosage of treatment and stored in MACS Tissue Storage Solution (Miltenyi Biotec, catalog No. 130-100-008). Afterward, the samples were dissected into tiny pieces (approximately 1 mm^3^) on ice and digested with reagents from a Mouse Tumor Dissociation Kit (Miltenyi Biotec, 130-096-730) in accordance with the package recommendations. After removal of the red blood cells, single cells were resuspended in ice-cold MACS buffer with CD45 Microbeads (Miltenyi Biotech, 130-110-618) and incubated for 15 min at 4°C before being washed with 2 mL of MACS buffer and centrifuged. Cells were filtered through a 30 μm cell strainer and passed through a prewetted MS column on a MACS magnetic cell separator (Miltenyi Biotech). The CD45^+^ cells were eluted with 1 mL of MACS buffer, and the cell number and viability were determined.

### Single-cell sequencing and data processing

Single-cell suspensions were adjusted to a cell concentration of 700–1,200 cells/μL, and libraries were constructed according to the instructions provided in the 10× Genomics Chromium Next GEM Single Cell 3ʹ Reagent Kits v3.1 (10× genomics, 1000268). The constructed libraries underwent sequencing using the Illumina Nova 6000 PE150 platform. The raw FASTQ files underwent processing using CellRanger software (Version 7.0.1), which included the incorporation of intronic reads. For the identification of potential doublets within individual samples, DoubletDetection software (Version 4.2) was applied, with the following parameters: boost_rate = 0.5 and voter_thresh = 0.9. Additionally, Scrublet software (Version 0.2.3) was employed, with a sim_doublet_ratio of 0.5, and the expected_doublet rate was evaluated based on the results obtained from DoubletDetection. Data integration and quality control procedures were conducted using Scanpy software (Version 1.9.3). Gene filtering was executed, involving the exclusion of genes expressed in fewer than three cells. Cell filtering incorporated the following three criteria: (i) The number of detected genes ranged from 500 to 8,000. (ii) The proportion of mitochondrial gene counts exceeded 10%. (iii) Cells that were not identified as doublets by both DoubletDetection and Scrublet were retained. Furthermore, single cells that coexpressed two sets of canonical cell type markers were categorized as doublets and subsequently removed. Ultimately, a total of 49,741 high-quality cells were retained for subsequent analysis.

### Dimensionality reduction and clustering

The gene expression levels of each cell were normalized to a count of 10,000 and log-transformed, preparing the data for subsequent analysis. Variable genes were selected independently for each sample using default parameters. The top 1,500 genes, characterized by the highest frequencies, were identified as the most variable genes, followed by the removal of ribosomal or mitochondrial genes. Cells with the most variable genes were then scaled with regression of total UMI counts, percent mitochondrial transcripts, S phase scores, and G_2_M phase scores. To eliminate any batch effects introduced by differences in mouse samples, the Harmony algorithm was employed. The scaled matrix was utilized to perform principal component analysis (PCA), which was subsequently adjusted into a Harmony matrix, treating samples as batches. The batch‐corrected matrix was employed to construct the nearest neighbor graph, and the Leiden algorithm was used to find clusters. In the first round of clustering, major cell types, including NK cells, T cells, B cells, DCs, neutrophils, Mac/Monos, mast cells, and CD45-negative cells, were identified based on canonical markers (Ncr1 for NK cells; Cd3e for T cells; Cd79a for B cells; Flt3 for DCs; Csf3r for neutrophils, Msr1 for Mac/Monos, and Tpsb2 for mast cells). Subsequently, independent clustering was performed within each major cell type with a higher resolution using the method described above. Uniform manifold approximation and projection (UMAP) was employed for visualization throughout the analysis.

### Calculation of gene signature scores

The calculation of gene signature scores was conducted using the “score_genes” function in Scanpy, utilizing default parameters. These scores were computed as the average expression level of a specific set of genes subtracted from the average expression of a reference set of genes randomly sampled from the entire gene pool. Gene signature datasets were obtained from various sources: (i) neutrophil-associated signature gene sets were retrieved from the gene ontology (GO) database. (ii) IFN-related gene sets, including canonical STING targets, IFNα response genes, and NK-κB target genes, were sourced from previously published research. (iii) T-cell cytotoxicity-related genes were obtained from a separate study. (iv) Similarly, gene sets for assessing cell cycle states were adopted from a previously reported scoring system.

### Differential expression and pathway enrichment analysis

Differential expression analysis was conducted using the Wilcoxon rank-sum test to calculate *P* values, and the Benjamini-Hochberg method was applied for multiple testing corrections. Differentially expressed genes (DEGs) between two groups of cells were identified based on adjusted *P* values and log_2_FC, with a minimum expression threshold of 10% in either group of cells. For comparisons between cell types, genes with an adjusted *P* value less than 0.01 and a log_2_FC greater than 0.75 were considered DEGs. For comparisons between treatment groups, genes with an adjusted *P* value less than 0.05 and a log_2_FC greater than 0.25 were considered DEGs. To characterize IFN-response subpopulations, “CD4T Treg IFN response,” “CD4T memory IFN response,” “CD8T IFN response,” and “Mac antitumoral” were designated as IFN-response subpopulations and compared with other nonresponse subpopulations. GO enrichment analysis for biological processes and KEGG enrichment analysis were performed to uncover the biological implications of DEGs. The hypergeometric distribution was applied to calculate *P* values, followed by multiple testing corrections using the Benjamini-Hochberg method, all conducted using clusterProfiler software (Version 4.9.0.002). Categories with adjusted *P* values less than 0.05 were regarded as statistically significant.

### Trajectory inference of neutrophils

To comprehend the evolutionary dynamics of neutrophil subpopulations, particularly the differentiation potential of neutrophils from the Neu_protumoral state into the Neu_antitumoral state, trajectory inference was performed using Monocle2 software (Version 2.18.0). Integrated gene expression matrices were employed to construct a CellDataSet, with genes expressed in fewer than 10 cells being removed from consideration. The most variable genes among neutrophils, as mentioned earlier, were identified as functional marker genes. These markers were subsequently utilized for sorting and clustering in subsequent analyses. After the DDRTree dimension reduction method was applied, the branched expression analysis modeling (BEAM) algorithm was used to identify genes with branch-dependent expression patterns that exhibit differences between the fate 1 and fate 2 trajectories.

### Cell–cell interaction analysis

To comprehensively depict potential cell–cell interactions, CellChat software (Version 1.6.1) was employed to infer both the number and strength of significant interactions. This was achieved by assessing the expression of a receptor in one cell population and a ligand in another. Normalized counts were loaded and processed with a standard parameter set. Receptor–ligand interactions were screened using the CellChatDB.mouse database as a reference, along with the precompiled mouse protein–protein interactions (PPI. mouse) as a priori network information. The control and cGAS treatment groups were analyzed independently using standard parameters and subsequently merged to compare differences in interactions between the groups. Interactions with a *P* value less than 0.01 were considered statistically significant.

### Statistical analysis

Statistical analysis was performed using GraphPad Prism 9 software. Statistical analyses included two-sided unpaired *t* test and one-way ANOVA with Tukey’s multiple comparison tests, as appropriate. Survival was plotted for every group by the Kaplan-Meier method and compared by a two-sided log-rank (Mantel-Cox) test. All data are shown as the mean ± standard error of the mean (SEM). *P* values of less than 0.05 were considered to indicate statistical significance, and the exact values are labeled in the results.

## Supplementary data

Supplementary data is available at *Protein & Cell Journal* online https://doi.org/10.1093/procel/pwae015.

pwae015_suppl_Supplementary_Figures_S1-S12

## Data Availability

The main data supporting the results in the study are available within the paper and its supplementary information. The raw and processed datasets generated during the current study are available for research purposes from the corresponding authors upon reasonable request.

## References

[CIT0001] Banskota S , Raguram A, Suh S et al. Engineered virus-like particles for efficient *in vivo* delivery of therapeutic proteins. Cell 2022;185:250–265.e16. 35021064 10.1016/j.cell.2021.12.021 PMC8809250

[CIT0002] Beilstein F , Abou Hamdan A, Raux H et al. Identification of a pH-sensitive switch in VSV-G and a crystal structure of the G pre-fusion state highlight the VSV-G structural transition pathway. Cell Rep 2020;32:108042. 32814045 10.1016/j.celrep.2020.108042

[CIT0003] Bulcha JT , Wang Y, Ma H et al. Viral vector platforms within the gene therapy landscape. Signal Transduct Target Ther 2021;6:53. 33558455 10.1038/s41392-021-00487-6 PMC7868676

[CIT0004] Cecchin R , Troyer Z, Witwer K et al. Extracellular vesicles: the next generation in gene therapy delivery. Mol Ther 2023;31:1225–1230. 36698310 10.1016/j.ymthe.2023.01.021 PMC10188631

[CIT0005] Chin EN , Sulpizio A, Lairson LL. Targeting STING to promote antitumor immunity. Trends Cell Biol 2023;33:189–203. 35931610 10.1016/j.tcb.2022.06.010

[CIT0006] Choi H , Kim Y, Mirzaaghasi A et al. Exosome-based delivery of super-repressor IkappaBalpha relieves sepsis-associated organ damage and mortality. Sci Adv 2020;6:eaaz6980. 32285005 10.1126/sciadv.aaz6980 PMC7141819

[CIT0007] Cui C , Chakraborty K, Tang XA et al. Neutrophil elastase selectively kills cancer cells and attenuates tumorigenesis. Cell 2021;184:3163–3177.e21. 33964209 10.1016/j.cell.2021.04.016 PMC10712736

[CIT0008] Dooley K , McConnell RE, Xu K et al. A versatile platform for generating engineered extracellular vesicles with defined therapeutic properties. Mol Ther 2021;29:1729–1743. 33484965 10.1016/j.ymthe.2021.01.020 PMC8116569

[CIT0009] Earley J , Piletska E, Ronzitti G et al. Evading and overcoming AAV neutralization in gene therapy. Trends Biotechnol 2023;41:836–845. 36503641 10.1016/j.tibtech.2022.11.006

[CIT0010] Escude Martinez de Castilla P , Tong L, Huang,C et al. . Extracellular vesicles as a drug delivery system: a systematic review of preclinical studies. Adv Drug Deliv Rev 2021;175:113801. 34015418 10.1016/j.addr.2021.05.011

[CIT0011] Ferreira JV , da Rosa Soares A, Ramalho J et al. LAMP2A regulates the loading of proteins into exosomes. Sci Adv 2022;8:eabm1140. 35333565 10.1126/sciadv.abm1140 PMC8956266

[CIT0012] Finkelshtein D , Werman A, Novick D et al. LDL receptor and its family members serve as the cellular receptors for vesicular stomatitis virus. Proc Natl Acad Sci U S A 2013;110:7306–7311. 23589850 10.1073/pnas.1214441110 PMC3645523

[CIT0013] Gao D , Wu J, Wu YT et al. Cyclic GMP-AMP synthase is an innate immune sensor of HIV and other retroviruses. Science 2013;341:903–906. 23929945 10.1126/science.1240933 PMC3860819

[CIT0014] Gao L , Sun Y, Zhang X et al. Wnt3a-loaded extracellular vesicles promote alveolar epithelial regeneration after lung injury. Adv Sci (Weinh) 2023;10:e2206606. 37072558 10.1002/advs.202206606 PMC10288279

[CIT0015] Goswami R , Jeon T, Nagaraj H et al. Accessing intracellular targets through nanocarrier-mediated cytosolic protein delivery. Trends Pharmacol Sci 2020;41:743–754. 32891429 10.1016/j.tips.2020.08.005 PMC7502523

[CIT0016] Gouveia MG , Wesseler JP, Ramaekers J et al. Polymersome-based protein drug delivery - quo vadis? Chem Soc Rev 2023;52:728–778. 36537575 10.1039/d2cs00106c PMC9890519

[CIT0017] Greening DW , Xu R, Ale A et al. Extracellular vesicles as next generation immunotherapeutics. Semin Cancer Biol 2023;90:73–100. 36773820 10.1016/j.semcancer.2023.02.002

[CIT0018] Gungabeesoon J , Gort-Freitas NA, Kiss M et al. A neutrophil response linked to tumor control in immunotherapy. Cell 2023;186:1448–1464.e20. 37001504 10.1016/j.cell.2023.02.032 PMC10132778

[CIT0019] Guo J , Huang L. Nanodelivery of cGAS-STING activators for tumor immunotherapy. Trends Pharmacol Sci 2022;43:957–972. 36089410 10.1016/j.tips.2022.08.006

[CIT0020] Hamilton JR , Tsuchida CA, Nguyen DN et al. Targeted delivery of CRISPR-Cas9 and transgenes enables complex immune cell engineering. Cell Rep 2021;35:109207. 34077734 10.1016/j.celrep.2021.109207 PMC8236216

[CIT0021] Han Z , Liu S, Pei Y et al. Highly efficient magnetic labelling allows MRI tracking of the homing of stem cell-derived extracellular vesicles following systemic delivery. J Extracell Vesicles 2021;10:e12054. 33489014 10.1002/jev2.12054 PMC7809601

[CIT0022] Hedrick CC , Malanchi I. Neutrophils in cancer: heterogeneous and multifaceted. Nat Rev Immunol 2022;22:173–187. 34230649 10.1038/s41577-021-00571-6

[CIT0023] Herrmann IK , Wood MJA, Fuhrmann G. Extracellular vesicles as a next-generation drug delivery platform. Nat Nanotechnol 2021;16:748–759. 34211166 10.1038/s41565-021-00931-2

[CIT0024] Hirschhorn D , Budhu S, Kraehenbuehl L et al. T cell immunotherapies engage neutrophils to eliminate tumor antigen escape variants. Cell 2023;186:1432–1447.e17. 37001503 10.1016/j.cell.2023.03.007 PMC10994488

[CIT0025] Hou X , Zaks T, Langer R et al. Lipid nanoparticles for mRNA delivery. Nat Rev Mater 2021;6:1078–1094. 34394960 10.1038/s41578-021-00358-0 PMC8353930

[CIT0026] Hu S , Fang Y, Chen X et al. cGAS restricts colon cancer development by protecting intestinal barrier integrity. Proc Natl Acad Sci U S A 2021;118:e2105747118. 34074794 10.1073/pnas.2105747118 PMC8201956

[CIT0027] Ilahibaks NF , Ardisasmita AI, Xie S et al. TOP-EVs: technology of protein delivery through extracellular vesicles is a versatile platform for intracellular protein delivery. J Control Release 2023;355:579–592. 36746337 10.1016/j.jconrel.2023.02.003

[CIT0028] Islam MA , Xu Y, Tao W et al. Restoration of tumour-growth suppression *in vivo* via systemic nanoparticle-mediated delivery of PTEN mRNA. Nat Biomed Eng 2018;2:850–864. 31015614 10.1038/s41551-018-0284-0 PMC6486184

[CIT0029] Kalluri R , LeBleu VS. The biology, function, and biomedical applications of exosomes. Science 2020;367:eaau6977. 32029601 10.1126/science.aau6977 PMC7717626

[CIT0030] Kasala D , Hong J, Yun CO. Overcoming the barriers to optimization of adenovirus delivery using biomaterials: current status and future perspective. J Control Release 2021;332:285–300. 33626335 10.1016/j.jconrel.2021.02.018

[CIT0031] Kitajima S , Tani T, Springer BF et al. MPS1 inhibition primes immunogenicity of KRAS-LKB1 mutant lung cancer. Cancer Cell 2022;40:1128–1144.e8. 36150391 10.1016/j.ccell.2022.08.015 PMC9561026

[CIT0032] Kreitz J , Friedrich MJ, Guru A et al. Programmable protein delivery with a bacterial contractile injection system. Nature 2023;616:357–364. 36991127 10.1038/s41586-023-05870-7 PMC10097599

[CIT0033] Lee KM , Lin CC, Servetto A et al. Epigenetic repression of STING by MYC promotes immune evasion and resistance to immune checkpoint inhibitors in triple-negative breast cancer. Cancer Immunol Res 2022;10:829–843. 35561311 10.1158/2326-6066.CIR-21-0826 PMC9250627

[CIT0034] Li S , Luo M, Wang Z et al. Prolonged activation of innate immune pathways by a polyvalent STING agonist. Nat Biomed Eng 2021;5:455–466. 33558734 10.1038/s41551-020-00675-9 PMC8126516

[CIT0035] Linde IL , Prestwood TR, Qiu J et al. Neutrophil-activating therapy for the treatment of cancer. Cancer Cell 2023;41:356–372.e10. 36706760 10.1016/j.ccell.2023.01.002 PMC9968410

[CIT0036] Ling S , Yang S, Hu X et al. Lentiviral delivery of co-packaged Cas9 mRNA and a Vegfa-targeting guide RNA prevents wet age-related macular degeneration in mice. Nat Biomed Eng 2021;5:144–156. 33398131 10.1038/s41551-020-00656-y

[CIT0037] Liu B , Lee BW, Nakanishi K et al. Cardiac recovery via extended cell-free delivery of extracellular vesicles secreted by cardiomyocytes derived from induced pluripotent stem cells. Nat Biomed Eng 2018;2:293–303. 30271672 10.1038/s41551-018-0229-7 PMC6159913

[CIT0038] Liu S , Mahairaki V, Bai H et al. Highly purified human extracellular vesicles produced by stem cells alleviate aging cellular phenotypes of senescent human cells. Stem Cells 2019;37:779–790. 30811771 10.1002/stem.2996 PMC6767364

[CIT0039] Liu S , Wu M, Lancelot M et al. BMI1 enables extensive expansion of functional erythroblasts from human peripheral blood mononuclear cells. Mol Ther 2021;29:1918–1932. 33484967 10.1016/j.ymthe.2021.01.022 PMC8116606

[CIT0040] Lokugamage MP , Vanover D, Beyersdorf J et al. Optimization of lipid nanoparticles for the delivery of nebulized therapeutic mRNA to the lungs. Nat Biomed Eng 2021;5:1059–1068. 34616046 10.1038/s41551-021-00786-x PMC10197923

[CIT0041] Low JT , Chandramohan V, Bowie ML et al. Epigenetic STING silencing is developmentally conserved in gliomas and can be rescued by methyltransferase inhibition. Cancer Cell 2022;40:439–440. 35487217 10.1016/j.ccell.2022.04.009

[CIT0042] Ma D , Liu S, Lal B et al. Extracellular matrix protein tenascin c increases phagocytosis mediated by CD47 loss of function in glioblastoma. Cancer Res 2019;79:2697–2708. 30898840 10.1158/0008-5472.CAN-18-3125 PMC8218246

[CIT0043] Machtakova M , Therien-Aubin H, Landfester K. Polymer nano-systems for the encapsulation and delivery of active biomacromolecular therapeutic agents. Chem Soc Rev 2022;51:128–152. 34762084 10.1039/d1cs00686j

[CIT0044] Meric-Bernstam F , Sweis RF, Hodi FS et al. Phase I dose-escalation trial of MIW815 (ADU-S100), an intratumoral STING agonist, in patients with advanced/metastatic solid tumors or lymphomas. Clin Cancer Res 2022;28:677–688. 34716197 10.1158/1078-0432.CCR-21-1963

[CIT0045] Meric-Bernstam F , Sweis RF, Kasper S et al. Combination of the STING agonist MIW815 (ADU-S100) and PD-1 inhibitor spartalizumab in advanced/metastatic solid tumors or lymphomas: an open-label, multicenter, phase Ib study. Clin Cancer Res 2023;29:110–121. 36282874 10.1158/1078-0432.CCR-22-2235 PMC11188043

[CIT0046] Milone MC , O’Doherty U. Clinical use of lentiviral vectors. Leukemia 2018;32:1529–1541. 29654266 10.1038/s41375-018-0106-0 PMC6035154

[CIT0047] Morad G , Helmink BA, Sharma P et al. Hallmarks of response, resistance, and toxicity to immune checkpoint blockade. Cell 2021;184:5309–5337. 34624224 10.1016/j.cell.2021.09.020 PMC8767569

[CIT0048] Morshedi Rad D , Alsadat Rad M, Razavi Bazaz S et al. A comprehensive review on intracellular delivery. Adv Mater 2021;33:e2005363. 33594744 10.1002/adma.202005363

[CIT0049] Muttenthaler M , King GF, Adams DJ et al. Trends in peptide drug discovery. Nat Rev Drug Discov 2021;20:309–325. 33536635 10.1038/s41573-020-00135-8

[CIT0050] Nikolic J , Belot L, Raux H et al. Structural basis for the recognition of LDL-receptor family members by VSV glycoprotein. Nat Commun 2018;9:1029. 29531262 10.1038/s41467-018-03432-4 PMC5847621

[CIT0051] Nusse R , Clevers H. Wnt/beta-catenin signaling, disease, and emerging therapeutic modalities. Cell 2017;169:985–999. 28575679 10.1016/j.cell.2017.05.016

[CIT0052] Paunovska K , Loughrey D, Dahlman JE. Drug delivery systems for RNA therapeutics. Nat Rev Genet 2022;23:265–280. 34983972 10.1038/s41576-021-00439-4 PMC8724758

[CIT0053] Ponzetta A , Carriero R, Carnevale S et al. Neutrophils driving unconventional T cells mediate resistance against murine sarcomas and selected human tumors. Cell 2019;178:346–360.e244. 31257026 10.1016/j.cell.2019.05.047 PMC6630709

[CIT0054] Qin X , Yu C, Wei J et al. Rational design of nanocarriers for intracellular protein delivery. Adv Mater 2019;31:e1902791. 31496027 10.1002/adma.201902791

[CIT0055] Quail DF , Amulic B, Aziz M et al. Neutrophil phenotypes and functions in cancer: a consensus statement. J Exp Med 2022;219:e20220011. 35522219 10.1084/jem.20220011 PMC9086501

[CIT0056] Ren Q , Cheng Y, Lv J. Boronate building blocks for intracellular protein delivery. Adv Healthc Mater 2023;12:e2202049. 36366889 10.1002/adhm.202202049

[CIT0057] Routledge D , Scholpp S. Mechanisms of intercellular Wnt transport. Development 2019;146:dev176073. 31092504 10.1242/dev.176073

[CIT0058] Russell JO , Monga SP. Wnt/beta-catenin signaling in liver development, homeostasis, and pathobiology. Annu Rev Pathol 2018;13:351–378. 29125798 10.1146/annurev-pathol-020117-044010 PMC5927358

[CIT0059] Samson N , Ablasser A. The cGAS-STING pathway and cancer. Nat Cancer 2022;3:1452–1463. 36510011 10.1038/s43018-022-00468-w

[CIT0060] Sanchez-Navarro M. Advances in peptide-mediated cytosolic delivery of proteins. Adv Drug Deliv Rev 2021;171:187–198. 33561452 10.1016/j.addr.2021.02.003

[CIT0061] Schunk SJ , Floege J, Fliser D et al. WNT-beta-catenin signalling – a versatile player in kidney injury and repair. Nat Rev Nephrol 2021;17:172–184. 32989282 10.1038/s41581-020-00343-w

[CIT0062] Segel M , Lash B, Song J et al. Mammalian retrovirus-like protein PEG10 packages its own mRNA and can be pseudotyped for mRNA delivery. Science 2021;373:882–889. 34413232 10.1126/science.abg6155 PMC8431961

[CIT0063] Shae D , Becker KW, Christov P et al. Endosomolytic polymersomes increase the activity of cyclic dinucleotide STING agonists to enhance cancer immunotherapy. Nat Nanotechnol 2019;14:269–278. 30664751 10.1038/s41565-018-0342-5 PMC6402974

[CIT0064] Sheller-Miller S , Radnaa E, Yoo JK et al. Exosomal delivery of NF-kappaB inhibitor delays LPS-induced preterm birth and modulates fetal immune cell profile in mouse models. Sci Adv 2021;7:eabd3865. 33523942 10.1126/sciadv.abd3865 PMC10671068

[CIT0065] Shirley JL , de Jong YP, Terhorst C et al. Immune responses to viral gene therapy vectors. Mol Ther 2020;28:709–722. 31968213 10.1016/j.ymthe.2020.01.001 PMC7054714

[CIT0066] Silva AM , Lazaro-Ibanez E, Gunnarsson A et al. Quantification of protein cargo loading into engineered extracellular vesicles at single-vesicle and single-molecule resolution. J Extracell Vesicles 2021;10:e12130. 34377376 10.1002/jev2.12130 PMC8329990

[CIT0067] Stewart MP , Sharei A, Ding X et al. *In vitro* and *ex vivo* strategies for intracellular delivery. Nature 2016;538:183–192. 27734871 10.1038/nature19764

[CIT0068] Tian Y , Tirrell MV, LaBelle JL. Harnessing the therapeutic potential of biomacromolecules through intracellular delivery of nucleic acids, peptides, and proteins. Adv Healthc Mater 2022;11:e2102600. 35285167 10.1002/adhm.202102600 PMC9232950

[CIT0069] Vargason AM , Anselmo AC, Mitragotri S. The evolution of commercial drug delivery technologies. Nat Biomed Eng 2021;5:951–967. 33795852 10.1038/s41551-021-00698-w

[CIT0070] Votteler J , Ogohara C, Yi S et al. Designed proteins induce the formation of nanocage-containing extracellular vesicles. Nature 2016;540:292–295. 27919066 10.1038/nature20607 PMC5729044

[CIT0074] Wang Q , Yu J, Kadungure T et al. ARMMs as a versatile platform for intracellular delivery of macromolecules. Nat Commun 2018;9:960. 29511190 10.1038/s41467-018-03390-x PMC5840177

[CIT0072] Wang F , Su H, Xu D et al. Tumour sensitization via the extended intratumoural release of a STING agonist and camptothecin from a self-assembled hydrogel. Nat Biomed Eng 2020;4:1090–1101. 32778697 10.1038/s41551-020-0597-7 PMC8848303

[CIT0071] Wang BZ , Luo LJ, Vunjak-Novakovic G. RNA and protein delivery by cell-secreted and bioengineered extracellular vesicles. Adv Healthc Mater 2022;11:e2101557. 34706168 10.1002/adhm.202101557 PMC8891029

[CIT0073] Wang L , Liu Y, Dai Y et al. Single-cell RNA-seq analysis reveals BHLHE40-driven pro-tumour neutrophils with hyperactivated glycolysis in pancreatic tumour microenvironment. Gut2023a;72:958–971. 35688610 10.1136/gutjnl-2021-326070 PMC10086491

[CIT0075] Wang Y , Xu M, Sun J et al. Glycolytic neutrophils accrued in the spleen compromise anti-tumour T cell immunity in breast cancer. Nat Metab2023b;5:1408–1422. 37563468 10.1038/s42255-023-00853-4

[CIT0076] Wei X , Zhang L, Yang Y et al. LL-37 transports immunoreactive cGAMP to activate STING signaling and enhance interferon-mediated host antiviral immunity. Cell Rep 2022;39:110880. 35649354 10.1016/j.celrep.2022.110880

[CIT0077] Wiklander OPB , Brennan MA, Lotvall J et al. Advances in therapeutic applications of extracellular vesicles. Sci Transl Med 2019;11:eaav8521. 31092696 10.1126/scitranslmed.aav8521 PMC7104415

[CIT0078] Wolf L , Boutros M. The role of Evi/Wntless in exporting Wnt proteins. Development 2023;150:dev201352. 36763105 10.1242/dev.201352 PMC10112924

[CIT0079] Yang K , Han W, Jiang X et al. Zinc cyclic di-AMP nanoparticles target and suppress tumours via endothelial STING activation and tumour-associated macrophage reinvigoration. Nat Nanotechnol 2022;17:1322–1331. 36302963 10.1038/s41565-022-01225-x

[CIT0080] Yang W , Mixich L, Boonstra E et al. Polymer-based mRNA delivery strategies for advanced therapies. Adv Healthc Mater 2023;12:e2202688. 36785927 10.1002/adhm.202202688 PMC11469255

[CIT0081] Yom-Tov N , Guy R, Offen D. Extracellular vesicles over adeno-associated viruses: Advantages and limitations as drug delivery platforms in precision medicine. Adv Drug Deliv Rev 2022;190:114535. 36210573 10.1016/j.addr.2022.114535

[CIT0082] Yonezawa A , Cavrois M, Greene WC. Studies of ebola virus glycoprotein-mediated entry and fusion by using pseudotyped human immunodeficiency virus type 1 virions: involvement of cytoskeletal proteins and enhancement by tumor necrosis factor alpha. J Virol 2005;79:918–926. 15613320 10.1128/JVI.79.2.918-926.2005 PMC538559

[CIT0083] You Y , Tian Y, Yang Z et al. Intradermally delivered mRNA-encapsulating extracellular vesicles for collagen-replacement therapy. Nat Biomed Eng 2023;7:887–900. 36635419 10.1038/s41551-022-00989-w

[CIT0084] Zhang X , Xu Q, Zi Z et al. Programmable extracellular vesicles for macromolecule delivery and genome modifications. Dev Cell 2020;55:784–801.e9. 33296682 10.1016/j.devcel.2020.11.007 PMC9719439

[CIT0085] Zheng W , Radler J, Sork H et al. Identification of scaffold proteins for improved endogenous engineering of extracellular vesicles. Nat Commun 2023;14:4734. 37550290 10.1038/s41467-023-40453-0 PMC10406850

[CIT0086] Zong Y , Lin Y, Wei T et al. Lipid nanoparticle (LNP) enables mRNA delivery for cancer therapy. Adv Mater 2023;35:e2303261. 37196221 10.1002/adma.202303261

