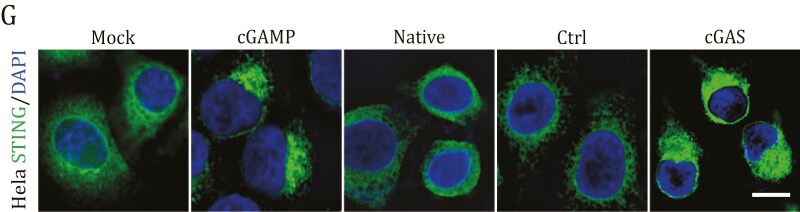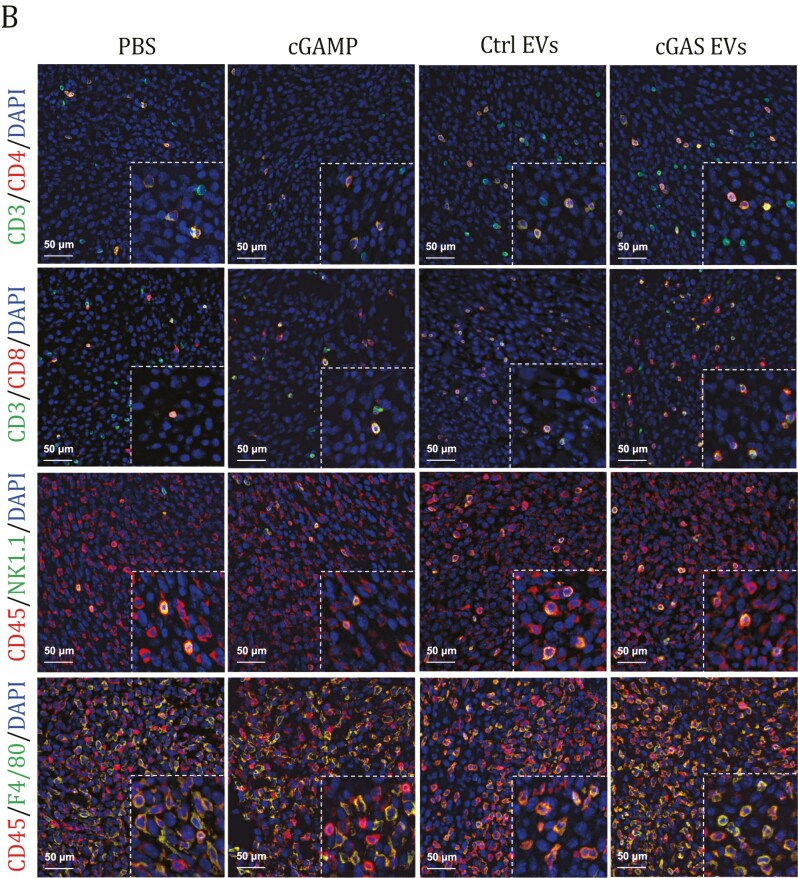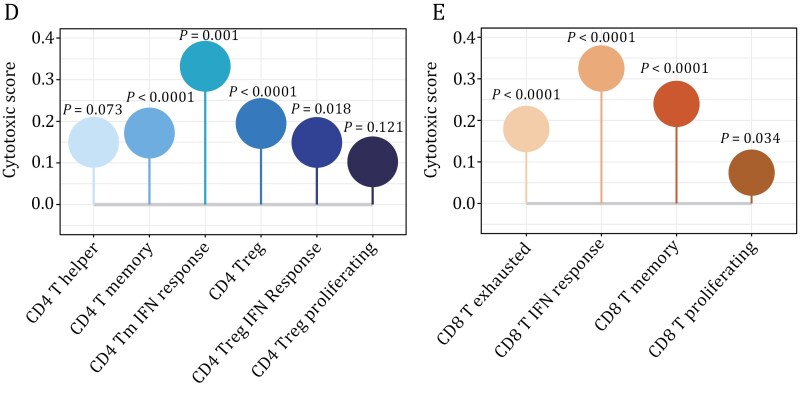# Correction to: Engineered extracellular vesicles enable high-efficient delivery of intracellular therapeutic proteins

**DOI:** 10.1093/procel/pwaf037

**Published:** 2025-06-06

This is a correction to: Ding Ma, An Xie, Jiahui Lv, Xiaolin Min, Xinye Zhang, Qian Zhou, Daxing Gao, Enyu Wang, Lei Gao, Linzhao Cheng, Senquan Liu, Engineered extracellular vesicles enable high-efficient delivery of intracellular therapeutic proteins, *Protein & Cell*, Volume 15, Issue 10, October 2024, Pages 724–743, https://doi.org/10.1093/procel/pwae015

The originally published version of this manuscript contained two errors that emerged during the compilation of Figs. 2G and 4B. In these cases, incorrect images were mistakenly incorporated into the Mock control group. Following a comprehensive and meticulous investigation, the authors identified these inadvertent errors, and the figures have been corrected online.

Moreover, Fig. 5 contained two typographical glitches—the absence of letters D and E—which occurred during the production stage. This figure has now also been corrected online.

These corrections do not in any way alter the interpretation or conclusions drawn in the paper. The authors wholeheartedly express their regret for any confusion or inconvenience caused by this unintended mishap.