# Supplementary material for: Engineered extracellular vesicles enable high-efficient delivery of intracellular therapeutic proteins
Source: Protein Cell. 2024 Mar 22;15(10):724–43. doi: 10.1093/procel/pwae015 (PMC11443452; doi:10.1093/procel/pwae015)
Supplement: pwae015_suppl_Supplementary_Figures_S1-S12 [file pwae015_suppl_supplementary_figures_s1-s12.pdf]

Figure S1

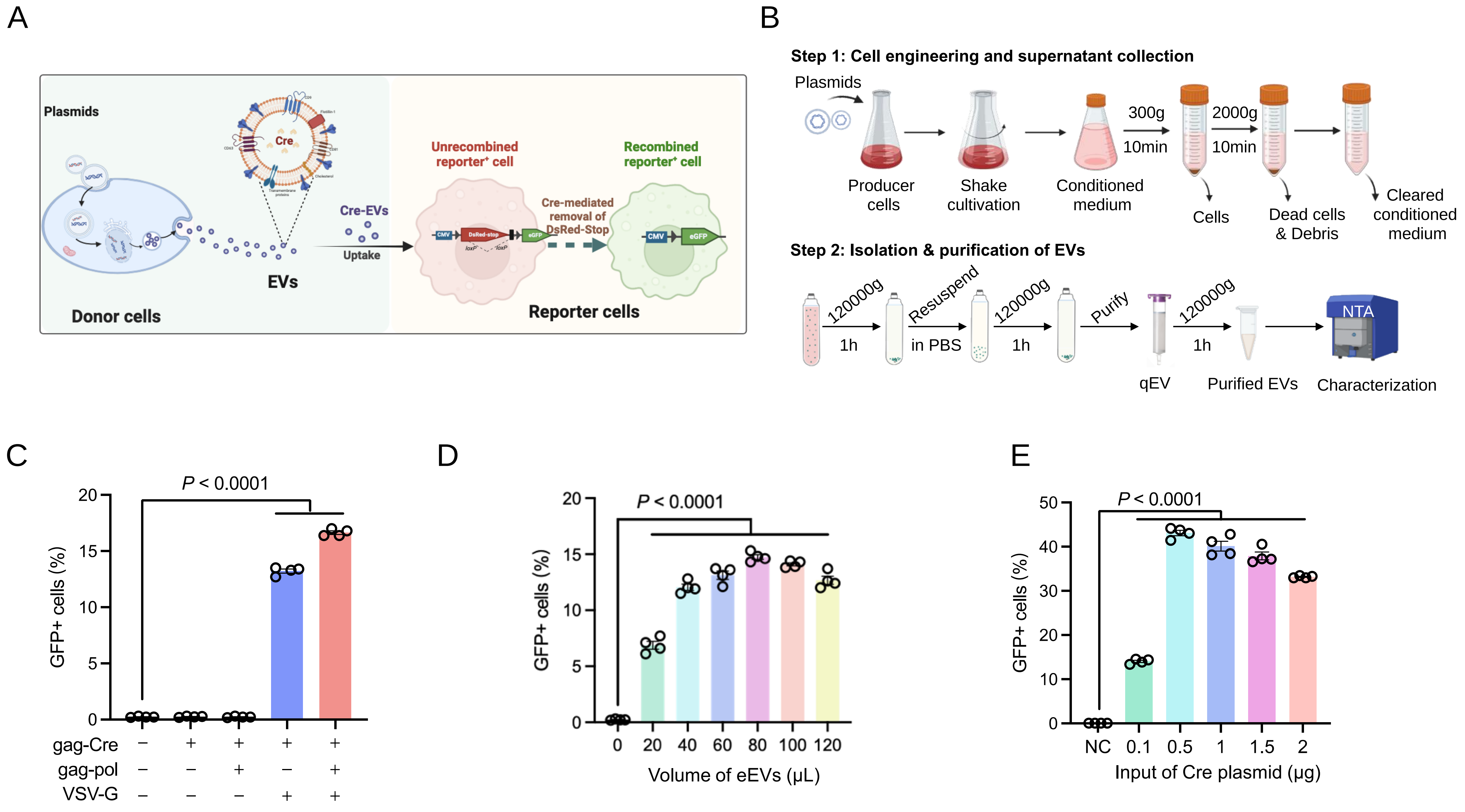

Figure S1. A Cre-loxP based reporter system for measuring nuclear delivery of Cre recombinase by engineered EVs or engineered virus-like particles (eVLPs). (A) Schematic showing the generation of engineered EVs or eVLPs for functional delivery of Cre recombinase into Cre-loxP reporter cells. (B) Outline of engineered EV and eVLP production. (C) Delivery of Cre recombinase mediated by eVLPs in the Cre-loxP GFP reporter cells. (D) Cre delivery efficiencies of eEVs at increasing doses, using the gag-Cre vector. (E) Cre-loxP recombination efficiencies based on direct DNA transfection to the same Cre-loxP reporter 293T cells by the gag-Cre vector. Statistical analyses were performed using a two-sided unpaired *t*-test. Data are the mean ± s.e.m. from 4 biological replicates.

Figure S2

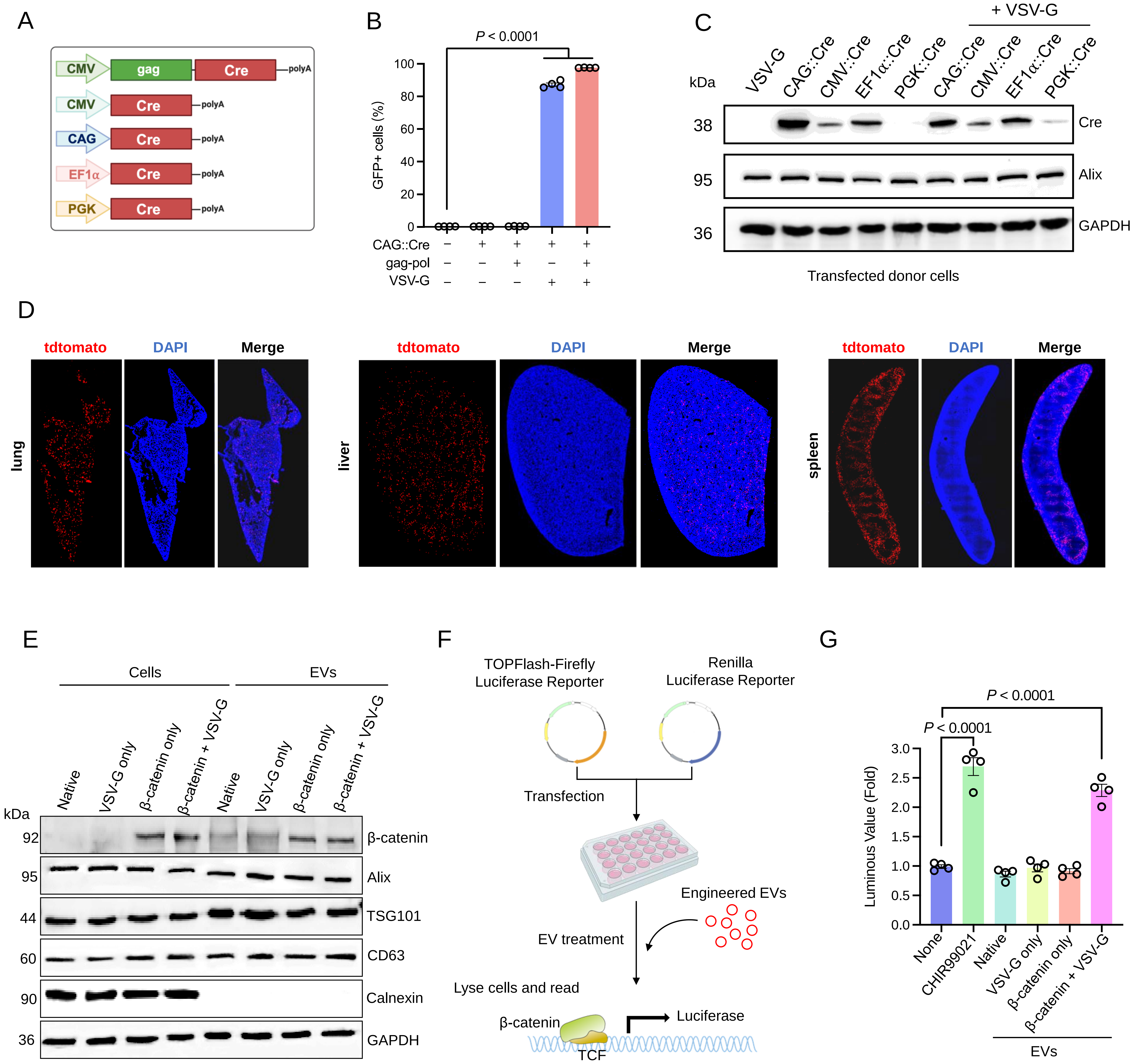

Figure S2. High-efficient transfer of Cre recombinase and β-catenin into recipient cells by an engineered extracellular vesicle (EV)-mediated delivery platform. (A) Schematic presentation of expression vectors encoding the cassette for Cre gene driven by the indicated promoter/enhancer for EV production in producer cells. (B) Production of EVs by co-transfection with CAG-Cre and VSV-G results in high-level Cre activity measured by percentages of GFP+ recipient cells, independent of gag-pol. (C) Western blot to validate the efficiency of Cre transgene expression in producer cells used for EV production. Of note, The CAG::Cre expression vector results in the highest level of Cre proteins in producer cells. (D) Representative immunofluorescence images of organs collected from Ai14 mice intraperitoneally administered with Cre EVs. (E) Western blot analysis of β-catenin and EV markers in the engineered donor cells and the corresponding EVs. (F) Schematic of the experimental design for assessment of β-catenin activity using the TOPFlash luciferase reporter assay. (G) Luciferase activity of multiple types of EVs. A small molecule CHIR99021 was used as a positive control for the assay. Statistical analyses were performed using two-sided unpaired *t*-test. Data are shown as the mean ± s.e.m. from 4 biological replicates.

Figure S3

A

Instrument Parameters		Measurement Parameters		Camera Control	
Temperature	25℃			Sensitivity	65
pH entered	7.0	Cell S/N	ZNTA	Frame Rate	30
Laser Wavelength	488nm			Shutter	150
Filter Wavelength	Scatter				

B

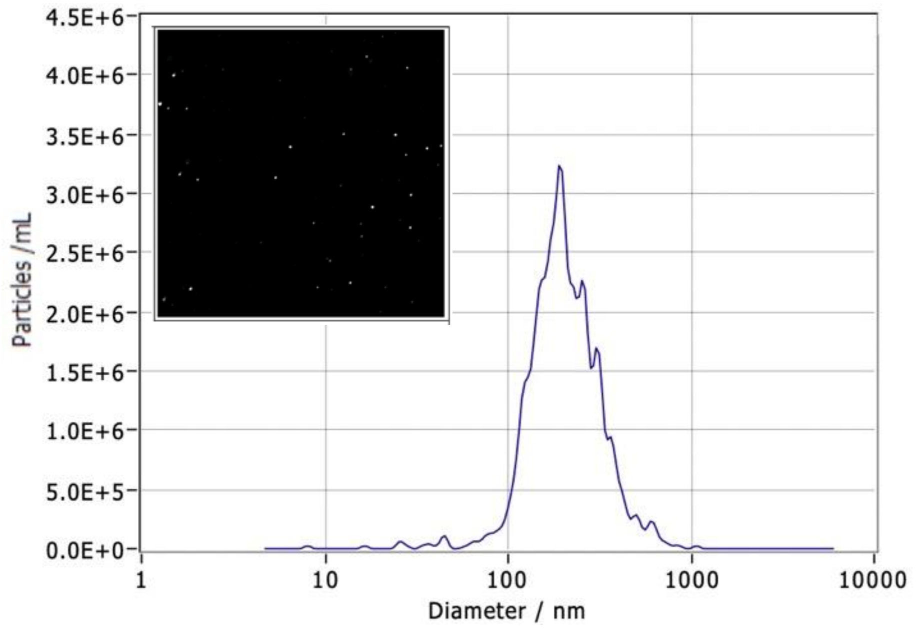

C D E

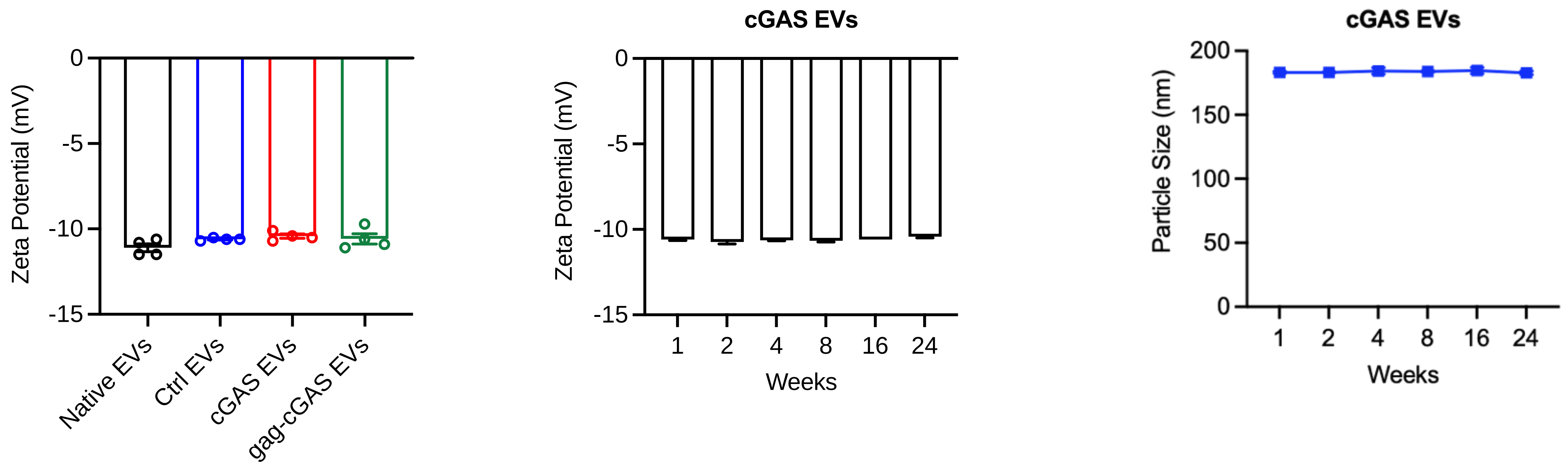

Figure S3. Characterization of various engineered EVs from producer cells. (A) Parameters of ZetaView settings for this study. (B) Representative images cropped from the analysis report. (C) Surface zeta potential of native EVs (n=4), Ctrl EVs (n=4), cGAS EVs (n=4) and gag-cGAS EVs (n=4) measured by dynamic light scattering. (D-E) Long-term stability of cGAS EVs stored at -80℃. No obvious changes in zeta potential (D) or particle size (E) were observed in PBS or FBS-containing media within 24 weeks. Data are presented as the mean ± s.e.m., n=3 independent experiments.

Figure S4

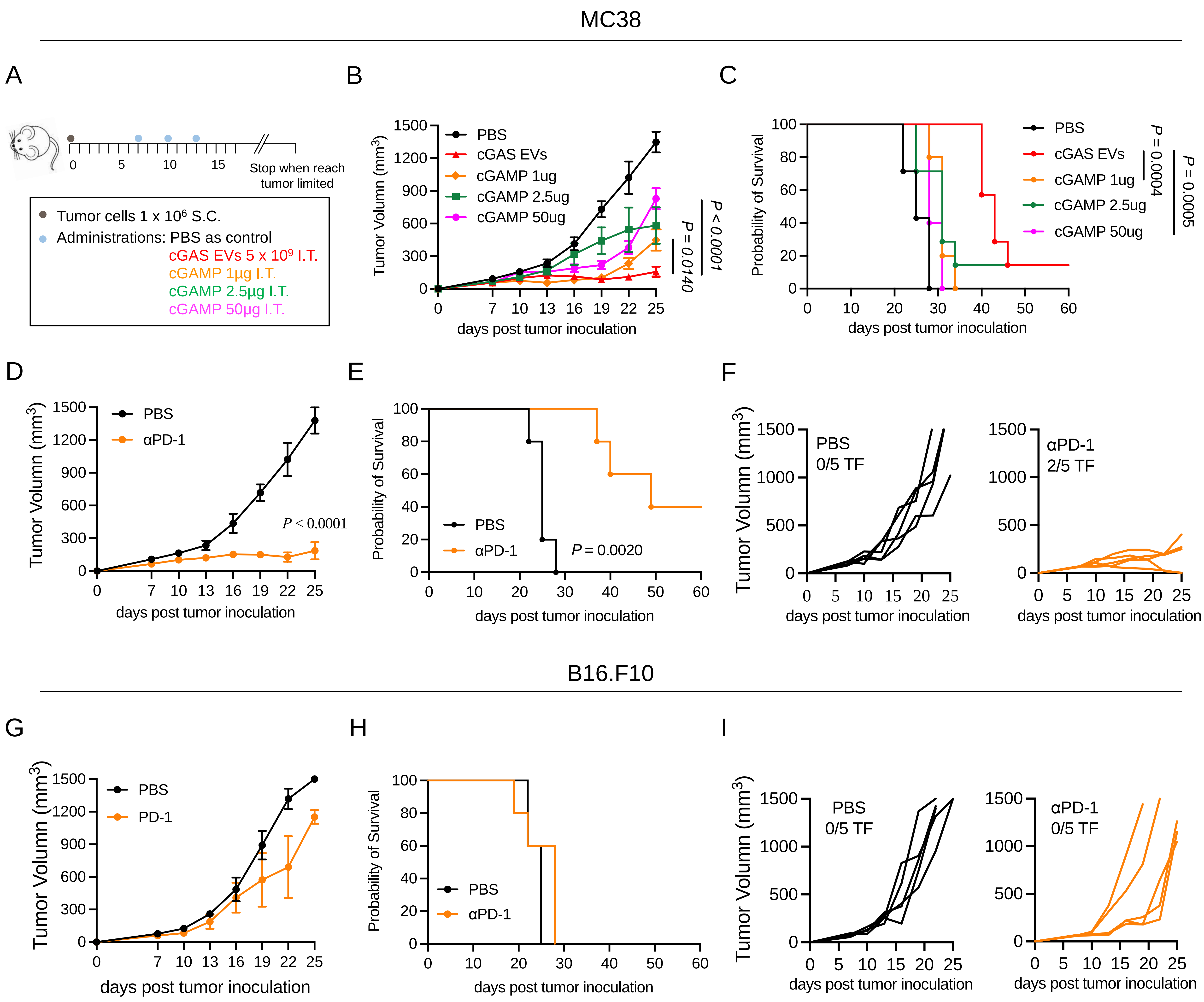

Figure S4. The anti-tumor effects of STING agonist (cGAMP) and immune checkpoint inhibitor in immune "hot" and "cold" tumor. (A) Schematic of tumor inoculation, treatment schedule, and time points for analysis of tumor growth. (B-C) MC38 tumor-bearing mice were intratumorally administration of PBS vehicle, cGAMP (1, 2.5, 50 μg), cGAS EVs ($5x10^9$ particles) at the indicated time points. Mean tumor volume (B), Kaplan-Meier survival curves (C) are shown. (D-I) C57BL/6 mice were s.c. inoculated with $1x10^6$ MC38 tumor cells (D-F) and B16.F10 tumor cells (G-I) in the right flank and then intraperitoneal injection of isotype control or anti-PD-1 antibody (200 μg) at day 7,10,13, respectively. Mean tumor volume (B, D, G), Kaplan-Meier survival curves (C, E, H), and spider plots of individual tumor growth curves (F, I) are shown. Statistical analyses among tumor volumes in B, D and G were performed using a two-sided unpaired *t*-test. Statistical analyses among survival curves in C, E and H were performed using two-sided log-rank (Mantel-Cox) test. Data are presented as the mean ± s.e.m.

Figure S5

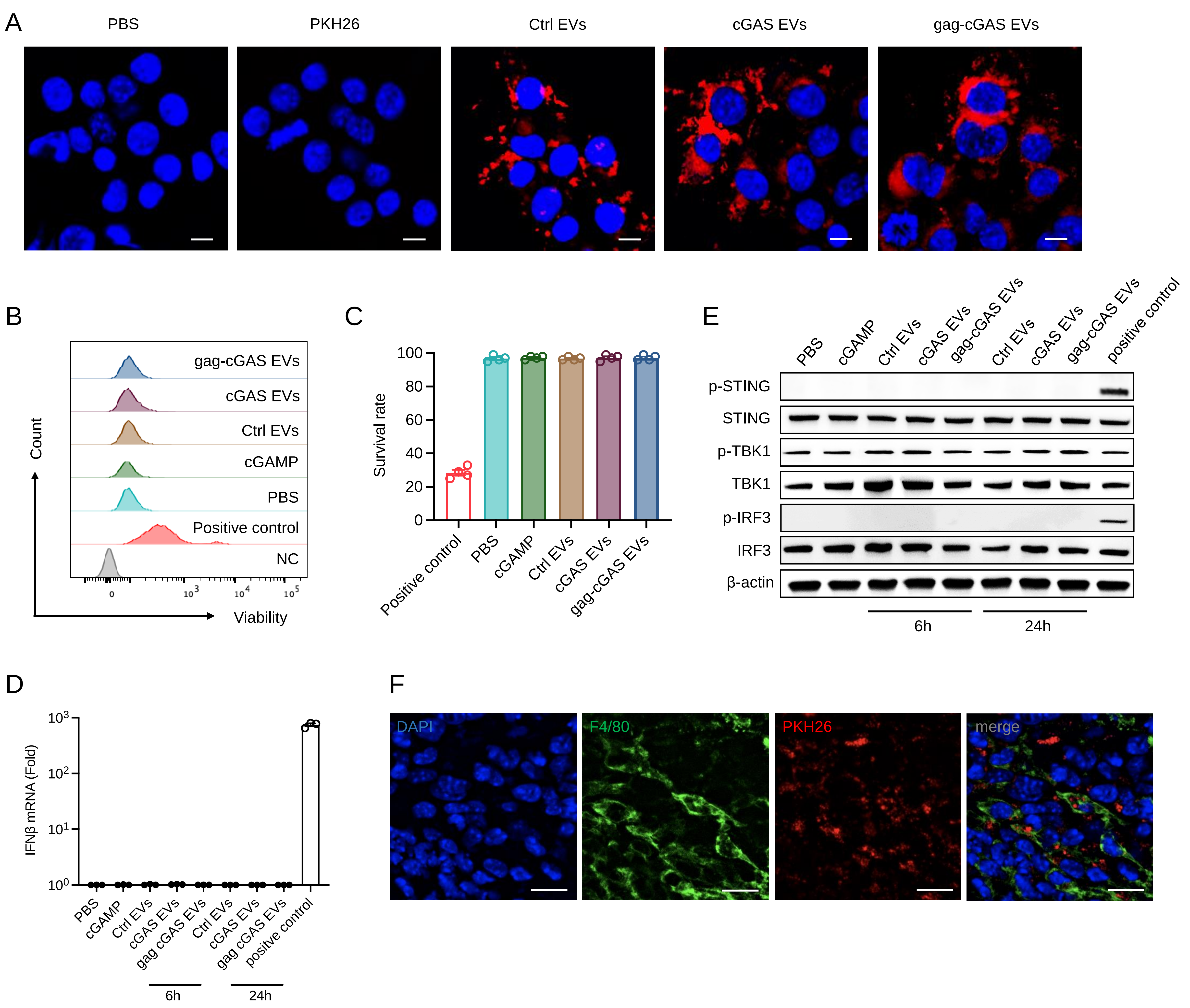

Figure S5. cGAS delivered by IDEA has no direct effect on tumor cells in vitro. (A) Representative images of PKH26-labelled EVs uptake by the murine colon cancer cell line MC38. scale bar 10 μm. (B-C) MC38 cells were incubated with different EVs for 6 h. Tumor cells were collected for apoptosis analysis by flow cytometry (B), and the survival rate (C) was calculated. (D) Relative *IFNβ* mRNA level in MC38 cells at 6, 24h. (E) Western blot analyses of p-STING, p-IRF3, p-TBK1, and their corresponding total protein in EV-treated MC38 cells at 6 h and 24 h. (F) Representative immunofluorescence images of F4/80^{+} macrophages after incubation with PKH26 labeled EVs. Scale bar, 20 μm.

Figure S6

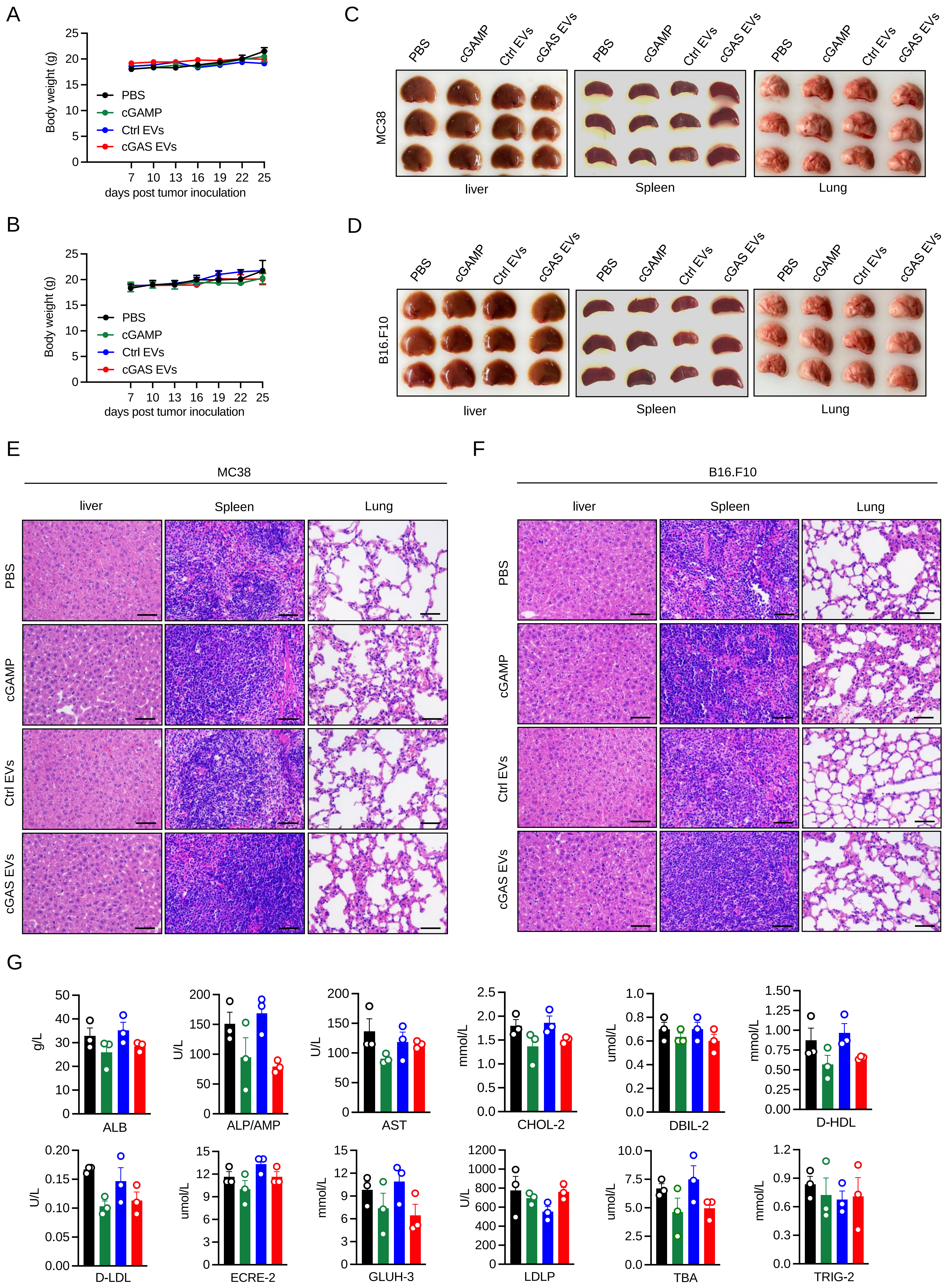

Figure S6. cGAS EVs are potent antitumor agents without detectable toxicity to mice. (A-B) Body weight changes of MC38 (A, n=7 mice) and B16.F10 tumor-bearing mice (B, n=6 mice) in different treatment groups are shown respectively. (C-D) Representative images of major organs collected from the last day of MC38 (C) and B16.F10 tumor-bearing mice (D) are shown. (E-F) H&E staining of major organs collected from MC38 (E) and B16.F10 tumor-bearing mice (F). (G) Serum of MC38 tumor bearing mice were collected for blood chemistry analysis once the tumor size endpoint was reached. Statistical significance in A, B and G was determined using ordinary one way ANOVA with Tukey's multiple comparisons, respectively. No statistical difference was detected between groups. Data are shown as the mean ± s.e.m. Scale bar, 50 μm.

Figure S7

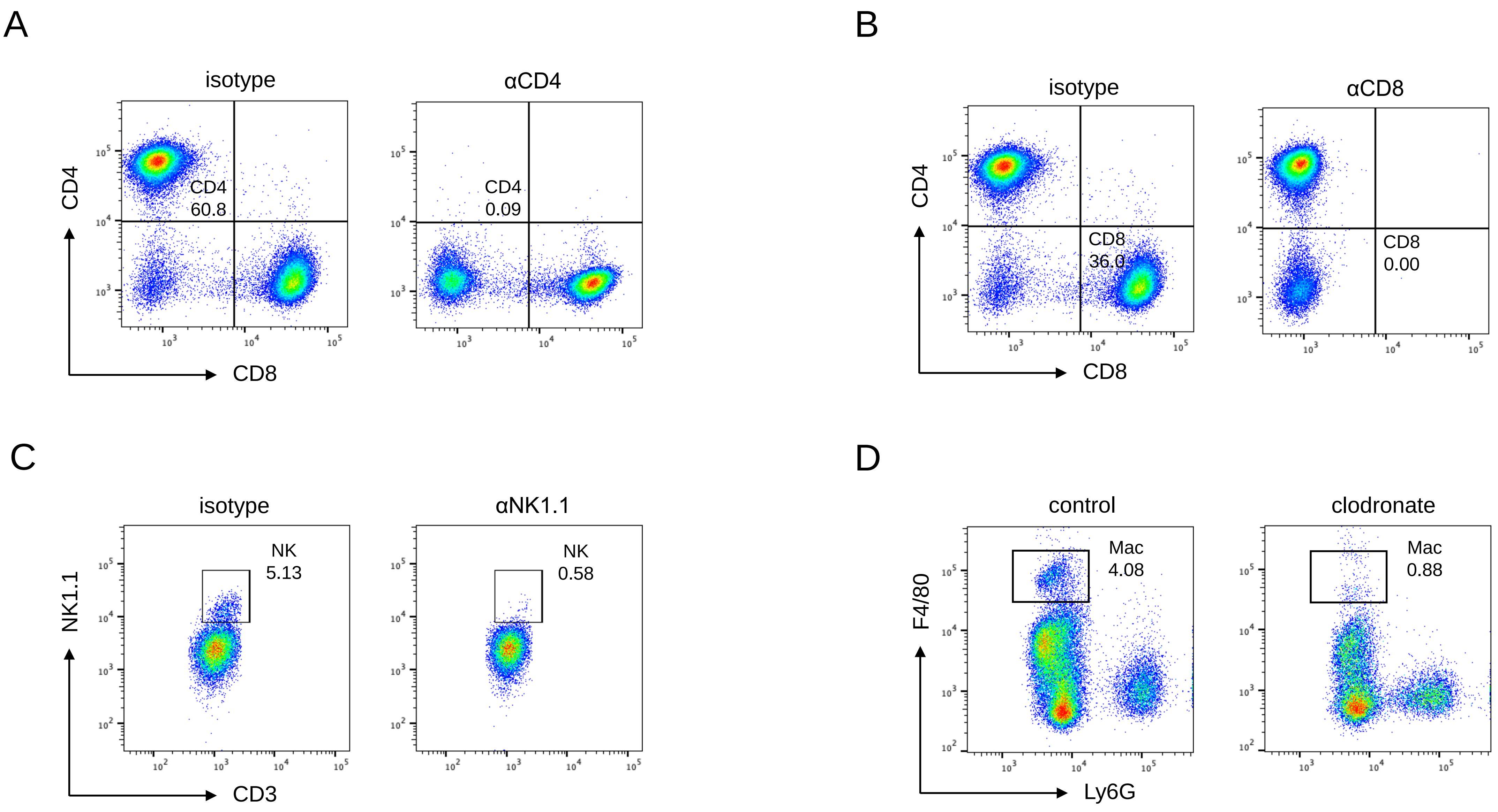

Figure S7. CD4⁺ T cells, CD8⁺ T cells and macrophages are critical to cGAS induced tumor growth inhibition. MC38 tumor-bearing C57BL/6 mice treated with cGAS EVs or along with antibodies of CD4⁺ T, CD8⁺ T, NK1.1 and clodronate at the indicated time points. Depletion of CD4⁺ T cells (A), CD8⁺ T cells (B), NK cells (C) and macrophages (D) were confirmed by flow cytometric analysis.

Figure S8

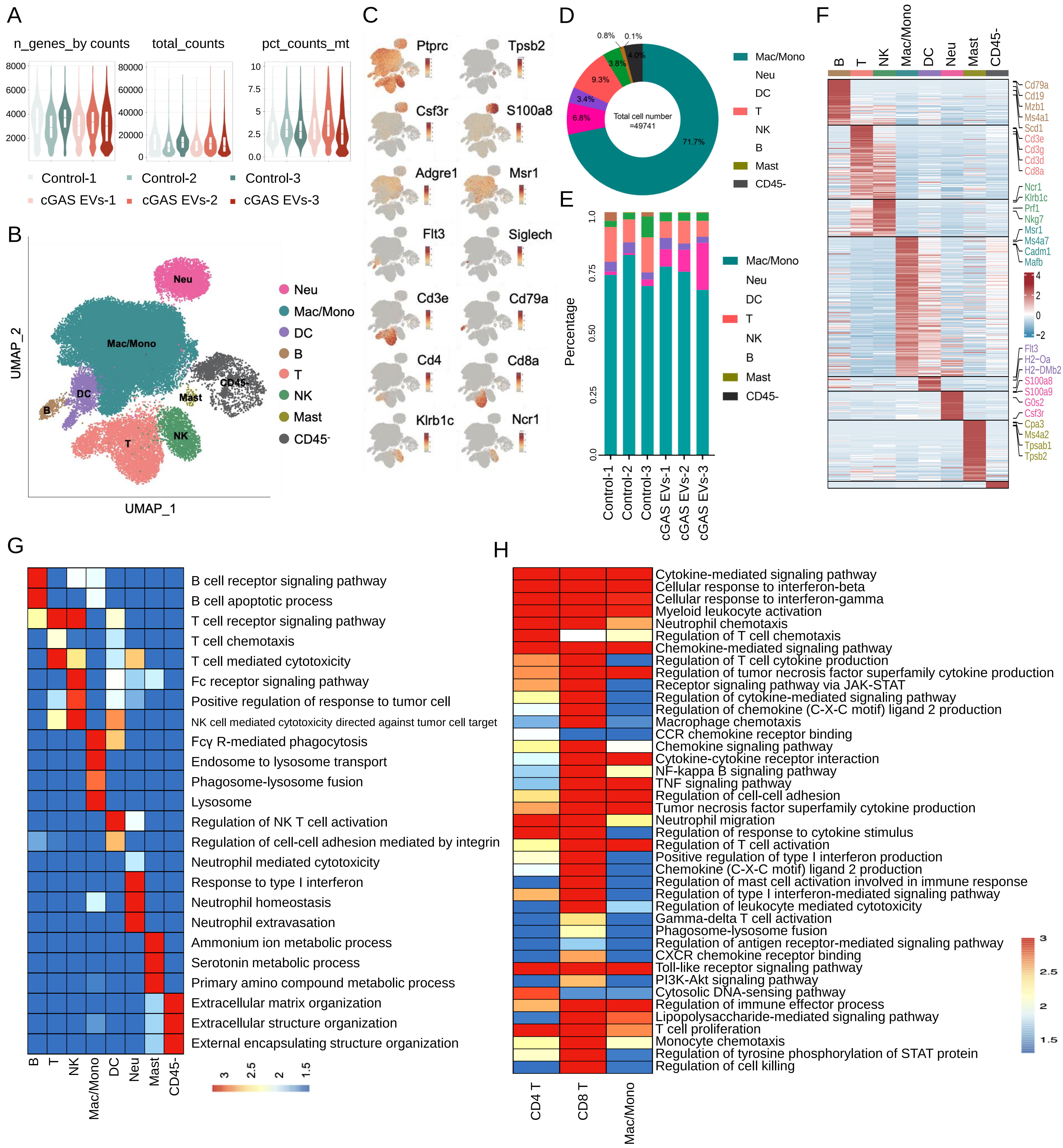

Figure S8. scRNA-seq analysis of cellular compositions in tumors. (A) Violin plots represent quality metrics of scRNA-seq data after filtering. The number of genes with at least 1 count in a cell, total number of counts for a cell and proportion of total counts for a cell that are mitochondrial are shown respectively. (B) Uniform manifold approximation and projection (UMAP) illustration of scRNA-seq data from total six mice colored by major cell populations. (C) UMAP projects of marker genes used to annotate cell types in B. (D) Pie chart depicting the total cell numbers and ratio of each cell population. (E) Stacked bar chart showing the proportion of each cell population in individual samples. (F) Heatmap of differentially expressed genes (including marker genes) in each cell population. Rows are ordered by hierarchical clustering and columns are grouped by cell types as denoted in the column annotation. (G) Heatmap depicting representative pathways across all cell populations. (H) Heatmap depicting selected activated pathways in IFN response subpopulations of $CD4^+$ T cells, $CD8^+$ T cells and macrophages.

Figure S9

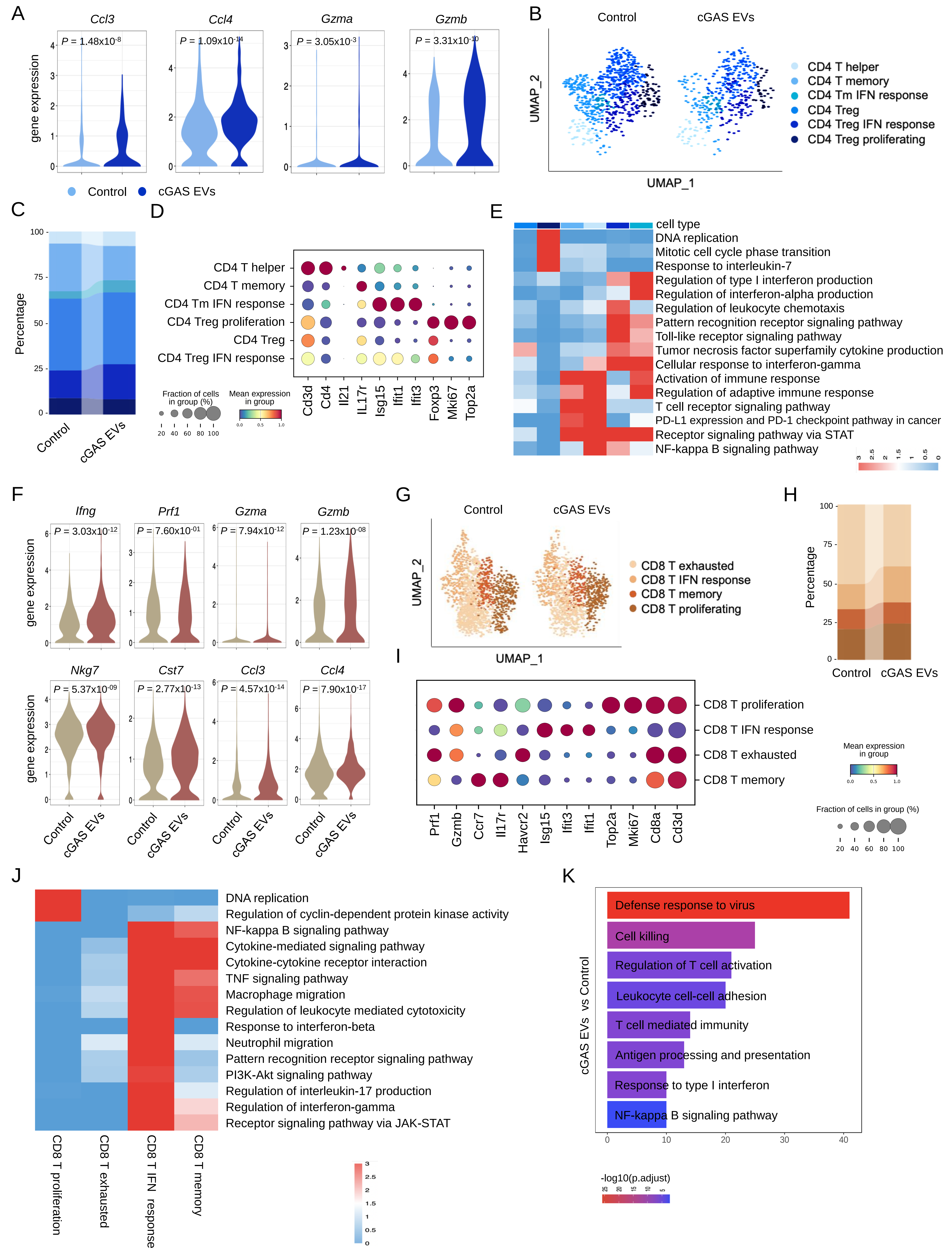
A
Ccl3
Ccl4
Gzma
Gzmb
P = 1.48x10-8
P = 1.09x10-14
P = 3.05x10-3
P = 3.31x10-10
gene expression
Control
cGAS EVs
B
Control
cGAS EVs
UMAP_2
UMAP_1
CD4 T helper
CD4 T memory
CD4 Tm IFN response
CD4 Treg
CD4 Treg IFN response
CD4 Treg proliferating
C
Percentage
Control
cGAS EVs
D
CD4 T helper
CD4 T memory
CD4 Tm IFN response
CD4 Treg proliferation
CD4 Treg
CD4 Treg IFN response
Fraction of cells in group (%)
Mean expression in group
Cd3d
Cd4
Il21
IL17r
Isg15
Ifit1
Ifit3
Foxp3
Mki67
Top2a
E
cell type
DNA replication
Mitotic cell cycle phase transition
Response to interleukin-7
Regulation of type I interferon production
Regulation of interferon-alpha production
Regulation of leukocyte chemotaxis
Pattern recognition receptor signaling pathway
Toll-like receptor signaling pathway
Tumor necrosis factor superfamily cytokine production
Cellular response to interferon-gamma
Activation of immune response
Regulation of adaptive immune response
T cell receptor signaling pathway
PD-L1 expression and PD-1 checkpoint pathway in cancer
Receptor signaling pathway via STAT
NF-kappa B signaling pathway
F
Ifng
Prf1
Gzma
Gzmb
P = 3.03x10-12
P = 7.60x10-01
P = 7.94x10-12
P = 1.23x10-08
Nkg7
Cst7
Ccl3
Ccl4
P = 5.37x10-09
P = 2.77x10-13
P = 4.57x10-14
P = 7.90x10-17
gene expression
Control
cGAS EVs
G
Control
cGAS EVs
UMAP_2
UMAP_1
CD8 T exhausted
CD8 T IFN response
CD8 T memory
CD8 T proliferating
H
Percentage
Control
cGAS EVs
I
CD8 T proliferation
CD8 T IFN response
CD8 T exhausted
CD8 T memory
Prf1
Gzmb
Ccr7
Il17r
Havcr2
Isg15
Ifit3
Ifit1
Top2a
Mki67
Cd8a
Cd3d
Mean expression in group
Fraction of cells in group (%)
J
DNA replication
Regulation of cyclin-dependent protein kinase activity
NF-kappa B signaling pathway
Cytokine-mediated signaling pathway
Cytokine-cytokine receptor interaction
TNF signaling pathway
Macrophage migration
Regulation of leukocyte mediated cytotoxicity
Response to interferon-beta
Neutrophil migration
Pattern recognition receptor signaling pathway
PI3K-Akt signaling pathway
Regulation of interleukin-17 production
Regulation of interferon-gamma
Receptor signaling pathway via JAK-STAT
CD8 T proliferation
CD8 T exhausted
CD8 T IFN response
CD8 T memory
K
cGAS EVs vs Control
Defense response to virus
Cell killing
Regulation of T cell activation
Leukocyte cell-cell adhesion
T cell mediated immunity
Antigen processing and presentation
Response to type I interferon
NF-kappa B signaling pathway
-log10(p.adjust)

Figure S9. cGAS EVs change the intratumoral T-cell population. (A) Violin plots comparing the expression levels of selected cytotoxic genes between the control and cGAS EV-treated groups in $CD4^+$ T cells. (B) UMAP visualization of $CD4^+$ T cell subtypes. (C) Alluvium bar shows the percentage of each $CD4^+$ T cell subtype in the two groups. (D) Marker gene expression for each $CD4^+$ T cell subtypes, with dot color and size representing the average scaled expression and fraction of marker gene expression, respectively. (E) Heatmap depicting selected pathways across $CD4^+$ T cell subtypes. (F) Violin plots comparing the expression levels of selected cytotoxic genes between the control and cGAS EV-treated groups in $CD8^+$ T cells. (G) UMAP visualization of $CD8^+$ T cell subtypes. (H) Alluvium bar showing the proportion of each cell population in an individual sample. show the percentage of each $CD8^+$ T cell subtype in the control and cGAS EV-treated groups. (I) Marker gene expression for each $CD8^+$ T cell subtype, with dot color and size representing the average scaled expression value and fraction of marker gene expression, respectively. (J) Heatmap depicting representative activated pathways in each $CD8^+$ T cell subtype. (K) Gene Ontology (GO) and KEGG enrichment analysis show the pathways significantly enriched in $CD8^+$ T IFN response cells. Genes upregulated in the cGAS EV treated group (versus the control group) were collected for enrichment analysis. Statistical analyses in A and F were performed using two-sided unpaired Wilcoxon test.

Figure S10

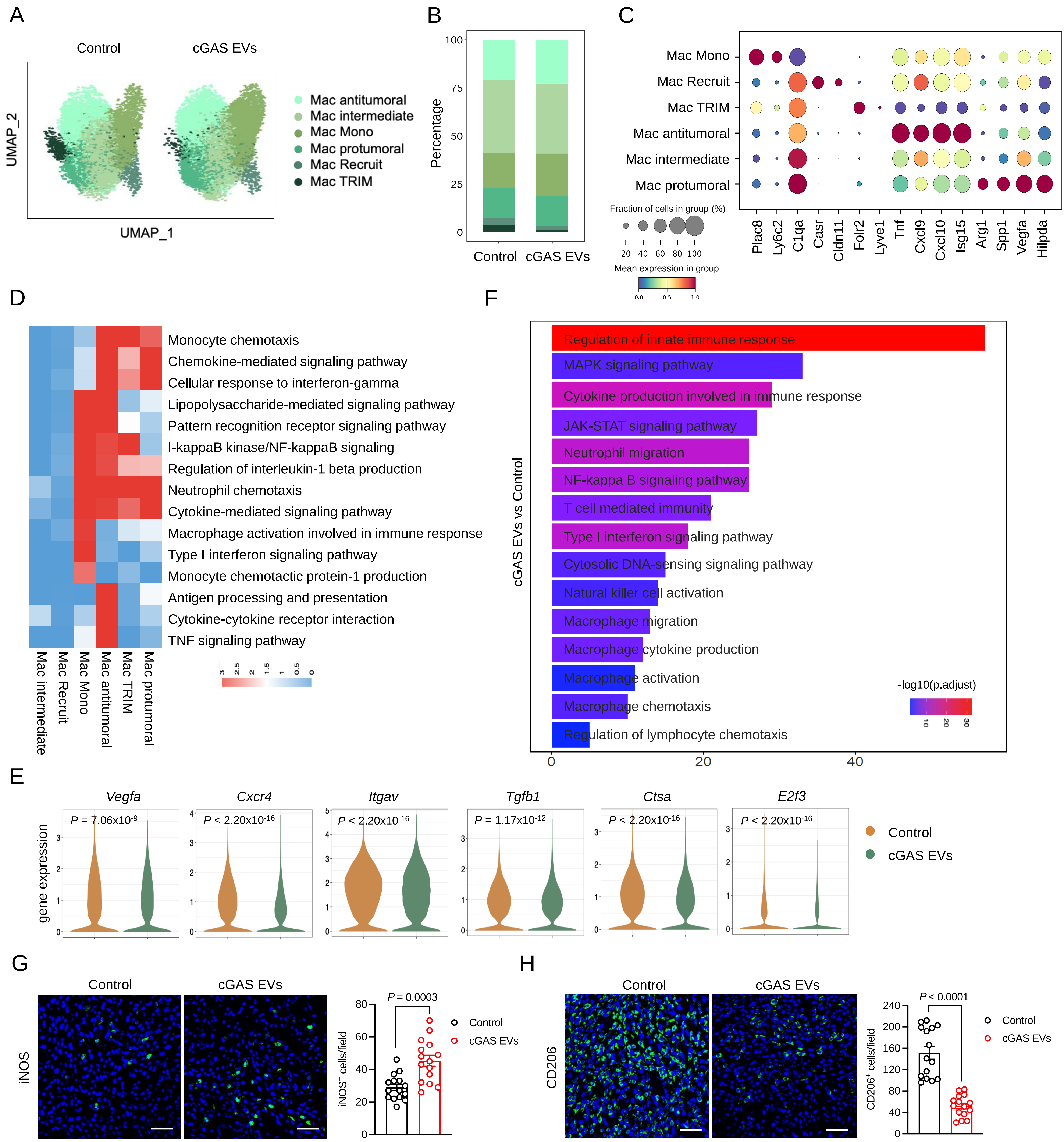

Figure S10. cGAS delivery converts macrophages from an immunosuppressive state to an immunogenic, tumoricidal state. (A) UMAP visualization of macrophage subtypes in the control and cGAS EV-treated group. (B) Alluvium bar show the percentage of each macrophage subpopulation in the control and cGAS EV-treated groups. (C) Marker gene expression for each macrophage subpopulation, with dot color and size representing the average scaled expression value and fraction of marker gene expression, respectively. (D) Heatmap depicting selected activation pathways in each macrophage subpopulation. (E) Violin plots comparing the expression level of selected protumoral genes in macrophages between the two groups. (F) GO and KEGG enrichment for macrophages. Genes upregulated in the cGAS EV-treated group (versus the control group) were collected for enrichment analysis. (G-H) Representative fluorescence images of M1-macrophages (G) and M2-macropahges (H) with statistical analysis of the number by manual counting. Scale bar, 50 μm. All images in G, H are representative of at least three biologically independent experiments. Statistical analyses in G, H was performed using a two-sided unpaired *t*-test. Statistical analyses in E were performed using a two-sided unpaired Wilcoxon test.

Figure S11

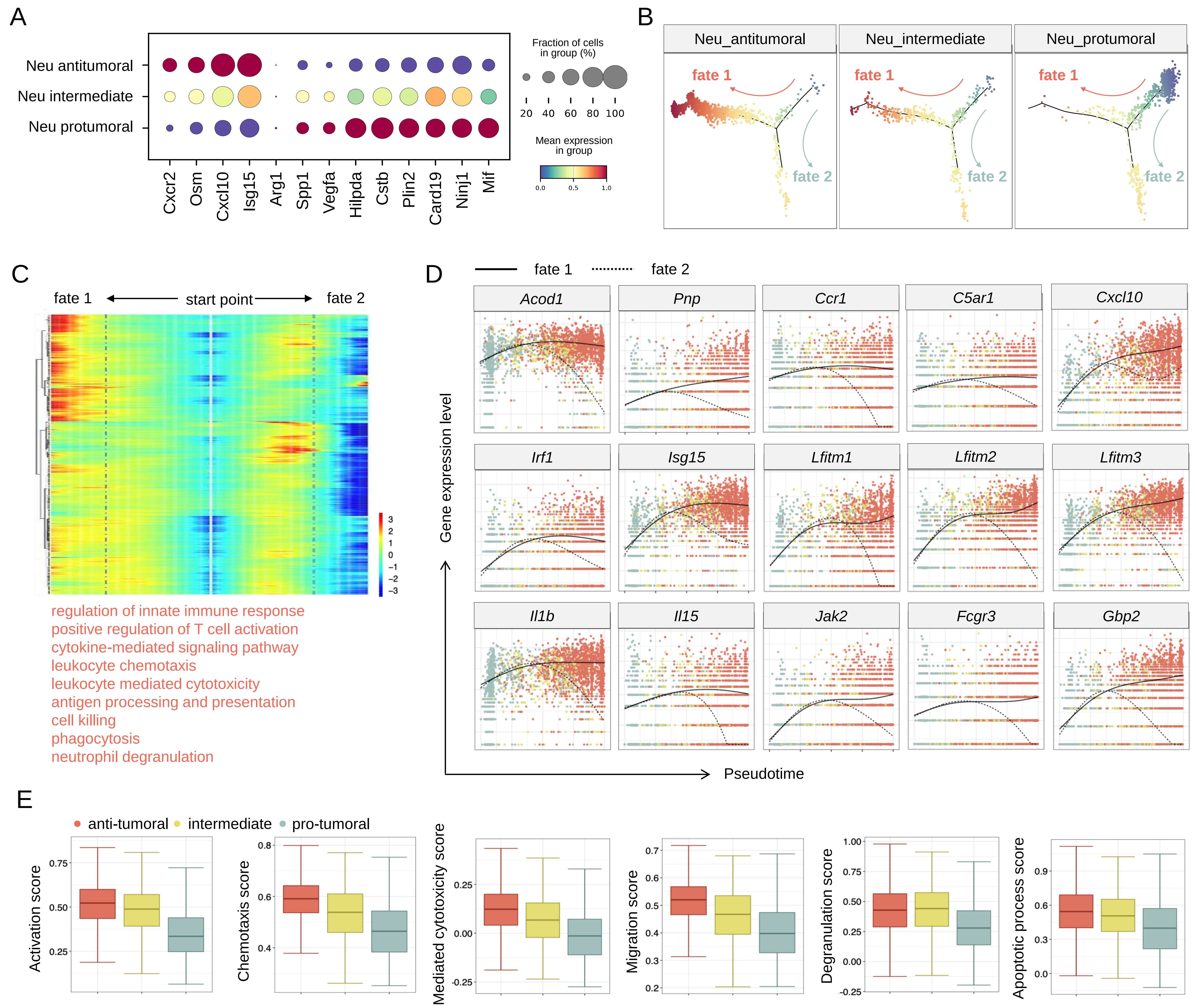

	antitumoral vs intermediate	antitumoral vs protumoral	intermediate vs protumoral		antitumoral vs intermediate	antitumoral vs protumoral	intermediate vs protumoral
activation	3.22x10-07	4.03x10-166	1.65x10-50	migration	4.14x10-23	1.78x10-146	1.31x10-22
chemotaxis	1.56x10-22	7.72x10-142	2.71x10-21	degranulation	3.15x10-01	3.27x10-58	1.36x10-31
cytotoxicity	5.40x10-17	4.17x10-124	2.97x10-23	apoptotic process	3.38x10-04	1.38x10-56	5.96x10-16

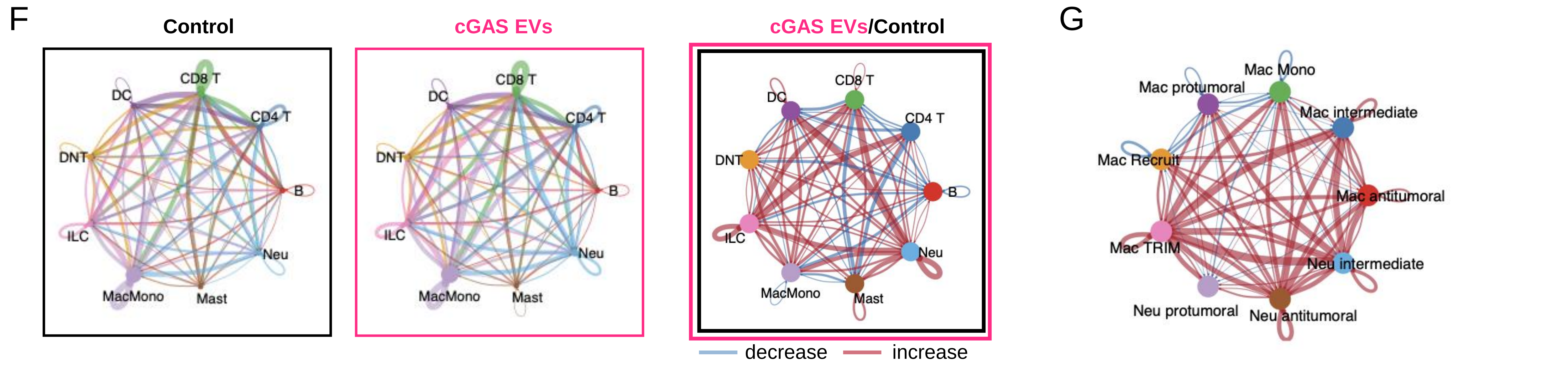

Figure S11. scRNA-seq analysis of the infiltrating neutrophil subpopulations. (A) Marker gene expression for each neutrophil subpopulation, with dot color and size representing the average scaled expression value and fraction of marker gene expression, respectively. (B) Monocle2 pseudotime trajectory of neutrophils colored by cluster identity. (C) Heatmap show the gene expression dynamics along the pseudotime trajectory and representative enriched pathways are shown. (D) Scatter plot show gene expression level which are significantly branch dependent. (E) Boxplots show the neutrophil-associated signature scores in 3 neutrophils subpopulations. (F) Circle plots show predicted ligand-receptor interactions network in control (left panel), cGAS EVs (middle panel), and comparison between two groups (right panel). (G) Circle plots show predicted ligand-receptor interactions network between neutrophils and macrophages. Statistical analyses in E were performed using two-sided unpaired Wilcoxon test.

Figure S12

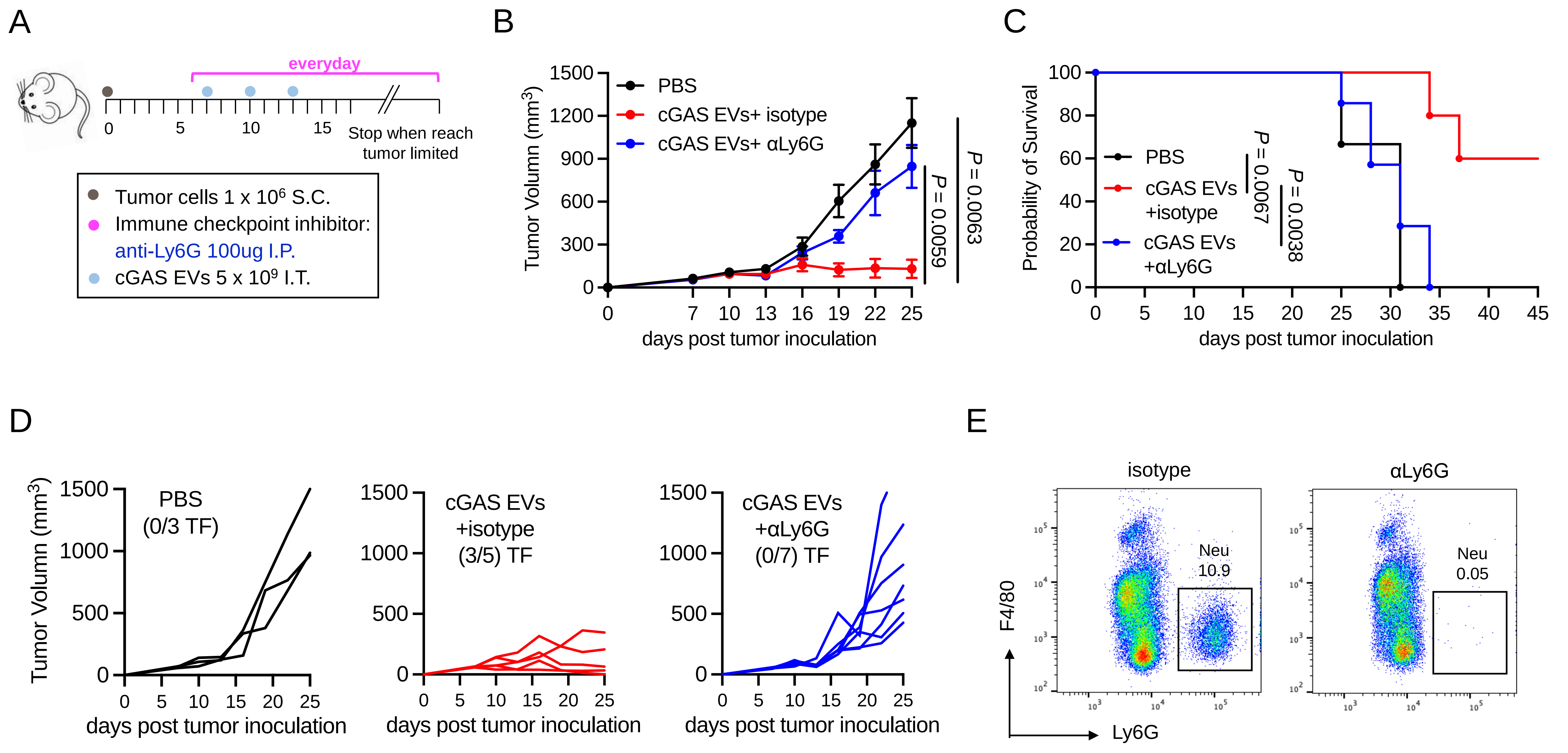

Figure S12. The essential role of neutrophils in cGAS induced anti-tumor immunity. (A-E) Schematic of tumor inoculation, treatment schedule, and time points for analysis of tumor growth. MC38 tumor-bearing C57BL/6 mice treated with PBS (n=3), cGAS EVs as indicated (n=5) and cGAS EVs along with neutrophils depletion antibody anti-Ly6G (n=7) as indicated (A) Mean tumor volume (B) Kaplan-Meier survival curves (C) spider plots of individual tumor growth curves (D) and the antibody depletion efficiency are shown (E) Statistical analyses among tumor volumes in B was performed using a two-sided unpaired *t*-test. Statistical analyses among survival curves in C were performed using two-sided log-rank (Mantel-Cox) test. Data are presented as the mean ± s.e.m.
